# Patent landscape of neglected tropical diseases: an analysis of worldwide patent families

**DOI:** 10.1186/s12992-017-0306-9

**Published:** 2017-11-14

**Authors:** Folahanmi Tomiwa Akinsolu, Vitor Nobre de Paiva, Samuel Santos Souza, Orsolya Varga

**Affiliations:** 1 Department of Preventive Medicine, Faculty of Public Health, University of Debrecen, Debrecen, Hungary; 2 Federal University of Rio Grande do Norte, Natal, Brazil; 3 Federal University of the Jequitinhonha and Mucuri Valleys, Natal, Brazil

**Keywords:** Neglected tropical diseases, Patents, Preventive chemotherapy, Intensified disease management, Mass drug administration

## Abstract

**Background:**

“Neglected Tropical Diseases” (NTDs) affect millions of people in Africa, Asia and South America. The two primary ways of strategic interventions are “preventive chemotherapy and transmission control” (PCT), and “innovative and intensified disease management” (IDM). In the last 5 years, phenomenal progress has been achieved. However, it is crucial to intensify research effort into NTDs, because of the emerging drug resistance. According to the World Health Organization (WHO), the term NTDs covers 17 diseases, namely buruli ulcer, Chagas disease, dengue, dracunculiasis, echinococcosis, trematodiasis, human African trypanosomiasis, leishmaniasis, leprosy, lymphatic filariasis, onchocerciasis, rabies, schistosomiasis, soil-transmitted helminthes, taeniasis, trachoma, and yaws.

The aim of this study is to map out research and development (R&D) landscape through patent analysis of these identified NTDs. To achieve this, analysis and evaluation have been conducted on patenting trends, current legal status of patent families, priority countries by earliest priority years and their assignee types, technological fields of patent families over time, and original and current patent assignees.

**Main body:**

Patent families were extracted from Patseer, an international database of patents from over 100 patent issuing authorities worldwide. Evaluation of the patents was carried out using the combination of different search terms related to each identified NTD.

In this paper, a total number of 12,350 patent families were analyzed. The main countries with sources of inventions were identified to be the United States (US) and China. The main technological fields covered by NTDs patent landscape are pharmaceuticals, biotechnology, organic fine chemistry, analysis of biological materials, basic materials chemistry, and medical technology. Governmental institutions and universities are the primary original assignees.

Among the NTDs, leishmaniasis, dengue, and rabies received the highest number of patent families, while human African trypanosomiasis (sleeping sickness), taeniasis, and dracunciliasis received the least. The overall trend of patent families shows an increase between 1985 and 2008, and followed by at least 6 years of stagnation.

**Conclusion:**

The filing pattern of patent families analyzed undoubtedly reveals slow progress on research and development of NTDs. Involving new players, such as non-governmental organizations may help to mitigate and reduce the burden of NTDs.

**Electronic supplementary material:**

The online version of this article (10.1186/s12992-017-0306-9) contains supplementary material, which is available to authorized users.

## Background

In Africa, Asia and South America, a good number of the population adds to the hundreds of millions of people affected by “neglected tropical diseases” (NTDs). According to the World Health Organization (WHO), the term NTDs covers the following 17 diseases: buruli ulcer, Chagas disease, dengue, dracunculiasis (guinea-worm disease), echinococcosis, trematodiasis, human African trypanosomiasis (sleeping sickness), leishmaniasis, leprosy, lymphatic filariasis, onchocerciasis (river blindness), rabies, schistosomiasis, soil-transmitted helminthes, taeniasis, trachoma, and yaws [1]. Based on the efforts of few researchers (e.g. Prof. Peter Hotez, Prof. Alan Fenwick and Prof. Alan Fairlamb), the concept of an umbrella category of these diverse diseases was established in the aftermath of the Millennium Development Goals (2000). In 2010, the WHO launched its first report on NTDs defining the strategic approaches for reducing the burden of NTDs [2]. Two years later, a “roadmap” was published revealing the milestones set for 2015 and 2020. The “roadmap” specifies targets for the eradication, elimination and intensified control of identified NTDs [3, 4].

For NTDs, the two primary methods of interventions are “preventive chemotherapy and transmission control” (PCT) covering “mass drug administraion” (MDA), and “innovative and intensified disease management” (IDM). In case of PCT, global strategies and applicable tools are readily available [5]. The most important tool for control is the administration of inexpensive (usually donated) drugs to entire at-risk populations without prior individual diagnosis [6]. PCT allows the regular and coordinated administration of single dose medicines on a large scale for the treatment of dracunculiasis, leprosy, lymphatic filariasis, onchocerciasis, schistosomiasis, soil-transmitted helminthiases and trachoma. IDM focuses more on NTDs for which simple tools and treatments are not yet available in which wide scale prevention cannot be applied (eg. effective drug does not exist or the high risk population cannot be reached) [7]. Some of the NTDs addressed by itensified disease management include buruli ulcer, Chagas disease, human African trypanosomiasis (sleeping sickness), leishmaniasis and yaws (endemic treponematoses) [4].

The MDA program is part of the PCT, and it involves regular drug donations. Several pharmaceutical companies, such as Merck & Co., Pfizer, GlaxoSmith etc. have been donating key drugs to address NTDs since the mid-1980s. In the case of Merck & Co., there is a program to donate Mectizan® indefinitely to support the fight of onchoceriasis [8]. From a recent WHO report on “Unprecedented progress against NTDs”, 1 billion people have been treated for at least one NTD in 2015 alone [9]. In spite of the success of MDA programs, they are controversial and raise important issues for consideration, such as suboptimal coverage or lack of efficacy [10]. Another concern about MDA programs is that they have been linked to drug and insecticide resistance experienced in the veterinary field [11, 12]. In fact, resistance to drugs has been detected in many parasites highlighting the risk of developing drug resistance when a single drug is used and the drug pressure is high [13]. Thus, drug donation cannot ameliorate the deficiency of new chemical entities being researched and developed. Taking on these challenges, the London Declaration (2012) based on governments, charities, and pharmaceutical companies aims to facilitate research and development (R&D) beyond ensuring the necessary supply of drugs and other interventions for NTDs for which treatments already exist. Tables 1, 2 and 3 shows the global data on number of countries affected by NTDs, disease burden, major interventions including information on prevention, treatment, drug resistance and donation, and effectiveness of interventions.

**Table 1:** Global data of countries affected by NTDs, drugs donated, burden of each disease, and number of patent families

Neglected Tropical Diseases (PCT/IDM)	Number of countries	Disease burden			Interventions				Effectiveness of current interventions		Granted patent families/all patent families
		Incidence	Prevalence	DALYs	Prevention	Treatment	Drug Resistance	Drug Donated	Prevention	Treatment	
Buruli ulcer (IDM)	33	No data	No data	No data	There are currently no primary preventive measures that can be applied. The mode of transmission is not known and there is no vaccine.	Rifampicin and streptomycin	Yes [29]	No	N/A	High	103/322
						Rifampicin and clarithromycin	Yes [29]	No		High	
Chagas disease (IDM)	21	8404	6,653,578	236,100	Vector control is the most effective method of prevention. Blood screening is necessary to prevent infection through transfusion and organ transplantation.	Benznidazole and Nifurtimox	Yes [30]	No	Low	High	449/1658
Dengue	>100	86,257,710	4,729,962	1,892,200	The main method of prevention is to combat vector mosquitoes. The first dengue vaccine, Dengvaxia (CYD-TDV) by Sanofi Pasteur, was registered in several countries.	No specific drug to treat	No	No	Low	N/A	829/2879
Dracunculiasis (PCT)	3	No data	No data	No data	There is no vaccine to prevent. Prevention is possible through complex preventive strategies.	No specific drug to treat	No	No	High	N/A	15/63
Echinococcosis	Very few countries are completely free of these parasites	313,264	1,382,975	600,000	Prevention programs focus on deworming of dogs and sheep. In the case of cystic echinococcosis, control measures also include improved food inspection, slaughterhouse hygiene, and public education campaigns.	Percutaneous treatment of the hydatid cysts with PAIR (Puncture, Aspiration, Injection, Re-aspiration) technique	Yes [31]	No	High	Low	96/535
Food-borne trematodiases	75	No data	71,095,424	168,500	Veterinary public health measures and food safety practices and education are recommended to reduce the risk of infection. Triclabendazole/Praziquantel through MDA programs.	Triclabendazole/Praziquantel	Yes [32]	Yes	High	High	59/269

*PCT* Preventive chemotherapy and transmission control

*IDM* innovative and intensified disease management

*MDA* mass drug administration

*N/A* not applicable

*SAFE* **s**urgery for advanced disease, **a**ntibiotics to clear *Chlamydia trachomatis* infection, **f**acial cleanliness, and **e**nvironmental improvement to reduce transmission

Sources: 1) The disease burden disability adjusted life years (DALYS) (the sum of years lost due to premature death (YLLs) and years lived with disability (YLDs)), Incidence (the total number of cases of a given disease in a specified population at a designated time), and Prevalence (the number of new cases of a given disease during a given period in a specified population), values -2015 were obtained from Global Health Data Exchange [43] and [14], 2) The number of countries and drugs used (Preventive Chemotherapy and Transmission Control; and Innovative and Intensified Disease Management) were obtained from WHO fact sheets [44], 3) Data on number of patent families was retrieved from Patseer database, 4) Efficacy/effectiveness/efficiency notes were obtained from the Third WHO Report on Neglected Tropical Diseases [45]

**Table 2:** Global data of countries affected by NTDs, drugs donated, burden of each disease, and number of patent families

Neglected Tropical Diseases (PCT/IDM)	Number of countries	Disease burden			Preventive Chemotherapy/Intensified disease management				Effectiveness of current interventions		Total granted/patent families
		Incidence	Prevalence	DALYS	Prevention	Treatment	Resistance	Donated	Prevention	Treatment	
Human African trypanosomiasis (IDM)	13	7,013	10,687	202,400	Vector control and effective disease surveillance.	Pentamidine and Suramin (First stage treatment)	Yes [33]	No	Low	High	41/198
Leishmaniasis (IDM)	10	1,051,824	3,859,307	3,859,307	Vector control and effective disease surveillance. Social mobilization and strengthening partnerships.	Amphotericin B, Miltefosine, fluconazole, itraconazole	Yes [34]	No	Low	High/Low	740/2652
Leprosy(PCT)	136	57,405	514,203	31,000	BCG Vaccination	Multidrug therapy	Yes [35]	No	Low	High	522/2206
Lymphatic filariasis (PCT)	73	No data	38,464,150	2,075,000	Albendazole through MDA programs. Mosquito control is a supplemental strategy supported by WHO.	Albendazole with either ivemectin or diethylcarbamazine	Yes [36]	Yes	High	High	69/287
Onchocerciasis (PCT)	31	No data	15,531,530	1,135,700	Ivermectin through MDA programs. Vector control.	Ivermectin	Yes [37]	Yes	High	High	88/313
Rabies	150	18,312	704	931,600	Integrated bite case management, Preventive immunization (vaccination)	Post-exposure prophylaxis, Integrated bite case management	No	No	High	High	569/2694
Schistosomiasis (PCT)	78	No data	252,339,520	2,613,300	Praziquantel through MDA programs. Additionally, access to safe water, improved sanitation, hygiene education, and snail control.	Praziquantel	Yes [38]	Yes	High	High	321/1722
Soil-transmitted helminthes (PCT)	118	No data	761,893,771	3,378,300	Albendazole/Mebendazole through MDA programs. Health education and improvement in personal hygiene are essential components of prevention.	Albendazole/Mebendazole	Probably [39]	No	High	High	83/584

*PCT* Preventive chemotherapy and transmission control

*IDM* innovative and intensified disease management

*MDA* mass drug administration

*N/A* not applicable

*SAFE* **s**urgery for advanced disease, **a**ntibiotics to clear *Chlamydia trachomatis* infection, **f**acial cleanliness, and **e**nvironmental improvement to reduce transmission

Sources: 1) The disease burden disability adjusted life years (DALYS) (the sum of years lost due to premature death (YLLs) and years lived with disability (YLDs)), Incidence (the total number of cases of a given disease in a specified population at a designated time), and Prevalence (the number of new cases of a given disease during a given period in a specified population), values -2015 were obtained from Global Health Data Exchange [43] and [14], 2) The number of countries and drugs used (Preventive Chemotherapy and Transmission Control; and Innovative and Intensified Disease Management) were obtained from WHO fact sheets [44], 3) Data on number of patent families was retrieved from Patseer database, 4) Efficacy/effectiveness/efficiency notes were obtained from the Third WHO Report on Neglected Tropical Diseases [45]

**Table 3:** Global data of countries affected by NTDs, drugs donated, burden of each disease, and number of patent families

Neglected Tropical Diseases (PCT/IDM)	Number of countries	Disease burden			Preventive Chemotherapy/Intensified disease management				Effectiveness of current interventions		Total granted/patent families
		Incidence	Prevalence	DALYS	Prevention	Treatment	Resistance	Donated	Prevention	Treatment	
Taeniasis	>75	No data	No data	503,000	Praziquantel/Niclosamide through MDA, identification and treatment of cases, health education including hygiene and food safety, improved sanitation, improved pig husbandry, anthelmintic treatment of pigs, vaccination of pigs, Improved meat inspection and processing of meat products.	Praziquantel/Niclosamide	Yes [40]	Yes	High	High	48/231
Trachoma (PCT)	42	No data	3,557,122	279,200	Azithromycin through MDA programs SAFE strategy	Azithromycin, Tetracycline	Yes [41]	Yes	High	High	514/2094
Yaws (IDM)	13	No data	No data	No data	Azithromycin through MDA programs. Health education and improvement in personal hygiene are essential components of prevention.	Azithromycin Benzathine Penicillin	Probably [42]	No	High	High	203/880

*PCT* Preventive chemotherapy and transmission control

*IDM* innovative and intensified disease management

*MDA* mass drug administration

*N/A* not applicable

*SAFE* **s**urgery for advanced disease, **a**ntibiotics to clear *Chlamydia trachomatis* infection, **f**acial cleanliness, and **e**nvironmental improvement to reduce transmission

Sources: 1) The disease burden disability adjusted life years (DALYS) (the sum of years lost due to premature death (YLLs) and years lived with disability (YLDs)), Incidence (the total number of cases of a given disease in a specified population at a designated time), and Prevalence (the number of new cases of a given disease during a given period in a specified population), values −2015 were obtained from Global Health Data Exchange [43] and [14], 2) The number of countries and drugs used (Preventive Chemotherapy and Transmission Control; and Innovative and Intensified Disease Management) were obtained from WHO fact sheets [44], 3) Data on number of patent families was retrieved from Patseer database, 4) Efficacy/effectiveness/efficiency notes were obtained from the Third WHO Report on Neglected Tropical Diseases [45]

According to the Global Burden of Disease 2015 study [14], the overall disability-adjusted life-years (DALYs) (an important parameter of disease burden) between 2005 and 2015, due to NTDs substantially declined. For example, DALYs of human African trypanosomiasis, a disease targeted for elimination has been reduced by more than 70% since 2005. However, the epidemiological improvement is not uniform. For example, both total and age-standardised DALYs rates for dengue increased by more than 50%.

The aim of this study is to determine the trends of R&D on NTDs by performing a patent landscape analysis. Historically, patents encourage research by giving monopoly to inventors over invention for 20 years and disclosing these inventions for public use after this period of time. To obtain a patent, an inventor must file a patent application. A patent application does not automatically give the applicant a temporary right against infringement. A patent has to be granted for it to be effective and enforceable against infringement. Performing a patent landscape analysis is an established method for understanding R&D trends in the biomedical field. This is because innovations stemming from biomedical research possess a great potential for developments which are often subjected to patent filings [15]. Additionally, due to novel, user friendly data visualization technologies and publicly accessible patent databases, patent landscape analysis has become an available tool for researchers and stakeholders to investigate emerging areas and also to identify economically attractive research gaps [16]. Considering the wide variety of contents available in patents, they are essential source of information for technological analysis [17, 18]. Although, technical possibilities for creating patent landscapes improved a lot in the last years, today only a limited number of patent landscapes are addressing comprehensive questions on health topics [19].

This analysis addressed the patenting trends, current legal status of patents, priority countries by earliest priority years and their assignee types, technological fields of patent families over time, and lastly, original and current patent assignees in the last 30 years.

## Methods

Patent families have been extracted from Patseer, an international database of patents from over 100 patent issuing authorities worldwide [20]. Evaluation of the patent families has been carried out using the combination of different search terms related to each identified NTD. The final set of keywords is presented in Additional file 1. Keywords of each identified NTD (their synonyms and truncation to cover different endings, singular/plural etc.) have been obtained from the Medical Subject Headings (MeSH) database of the National Library of Medicine in which vocabulary thesaurus is used for indexing articles for PubMed, fact sheets relating to NTDs produced by the WHO, and Google Scholar.

For visualization purposes of R&D trends, an additional database and software, PatBase [21] was also used; the patent collection retrieved from Patseer was uploaded and analyzed in PatBase.

Technology domains and International Patent Codes (IPC) have been adopted for topic identification for each identified NTD. Technology domains are comprehensive allocations of patented inventions. The first 4 digits of IPC codes are linked with the 35 fields of technology, in which categorization has been revised by the World Intellectual Property Organization [22]. The IPC categorizes similar inventions thus, providing a single source to browsing through all inventions relating to a specific NTD using the titles, abstracts and claims of patent families have been accessed.

The analysis was based on simple patent families (a group of one or more patent applications which represent the same invention) since patent applications are often filed in more than one country. Duplicates have been removed by creating simple families which represent the family members of a particular patent record with same priority dates.

Legal status information is an important component of patent information, as it determines whether examination of a patent application is still pending, or the application was withdrawn or rejected, or a patent has been granted and it is still valid or a granted patent has expired, lapsed or been revoked due to an opposition. In PatSeer, setting “one member per family” deduplication mode for an entered query, the displayed record is represented by the legal status of its family members. For example, if any one of the family members has legal status as granted, the record displayed will be marked as granted.

## Results

**Fig. 1:**
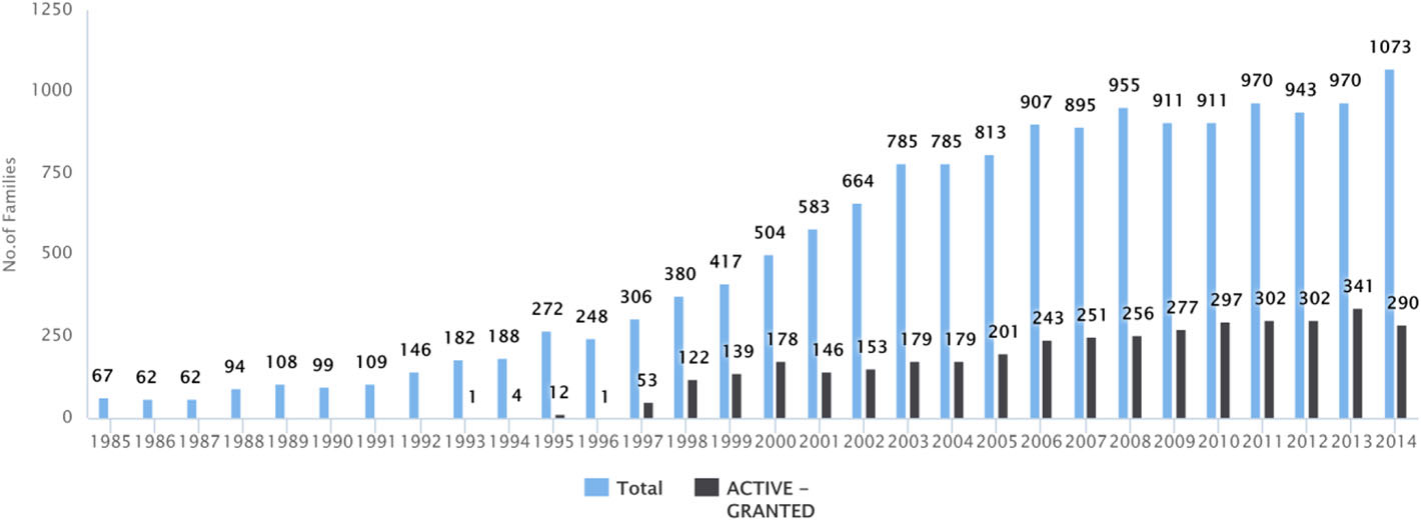
Patenting trend by number of granted patent families and the total patent families. The overall filing trend in the last 30 years for NTDs reveals an increasing trend between 1985 and 2014. Following the intense growing period between 1985 and 2008, there is no steady increase in the number of total patent families, but there is a slow but continous growth in the number of granted patent families. Patent applications are not published until after 18 months, this explains why no data is presented after 2014

In this study, the total number of patent families reviewed was 12,350, out of which 3179 were granted patent families. A distinction between research activities for each NTD has been observed. Among the NTDs, leishmaniasis, dengue, and rabies received the highest number of families, while taeniasis and dracunciliasis the least. The number of granted patent families and total patent families for each NTD is presented in Table 1. The overall patenting trend for NTDs is often characterized by the total number of simple families and granted patent families (by year when it was granted). As presented in Fig. 1, there is a substantial increase in patenting activities between 1985 and 2014 both in the total numbers of patent families including applications and in granted patent families. Although, total patenting activity was fluctuating between 2003 and 2008 which was followed by 6 years stagnation, mainly because of the decreasing number of applications. The increase in the granted families is continuous but slow.

The variable trends in NTDs patenting can be classified into three distinguished catergories. The first category shows an increasing trend in the number granted patents based on patent families (buruli ulcer, Chagas disease, dengue, onchocerciasis); the second category is mostly characterized by stagnation (echinococcosis, leishmaniosis, leprosy, rabies, schistosomiasis, trachoma, yaws); while the third category lacks a clear trend due to the low number of filings (dracunculiasis, food-borne trematodiasis, human African trypanosomiasis, lymphatic filariasis, soil-transmitted helminthes, taeniasis). There was no significant increase in the number of granted patent families for any of the NTDs in the last 10 years. The figures of annual patenting trends for each NTD are presented in Additional file 2.

While the biphasic trends of IDM and PCT diseases appear to be similar, the patenting trends of these two groups reveal a slight noticable difference. In comparison with the PCT group, the IDM group show a more intense growth period and stagnation after 2008 (see Additional file 2).

**Fig. 2:**
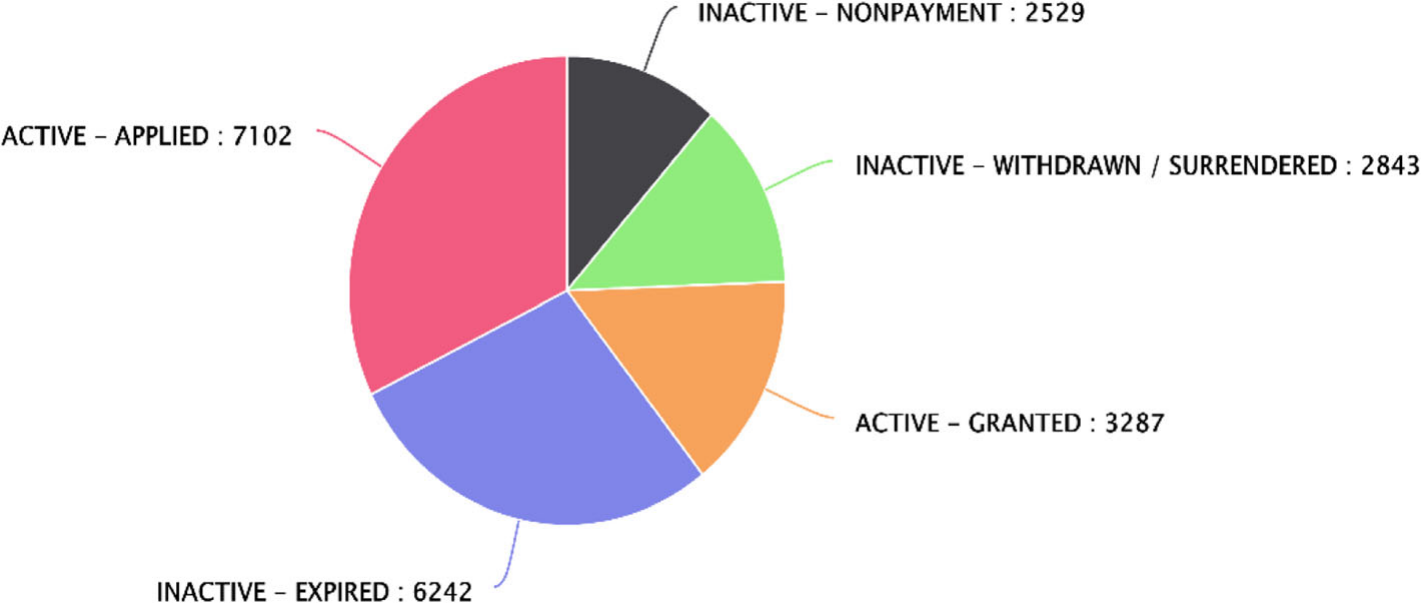
Current legal status of patent families of NTDs. Almost 50% of the patents are not active. Record numbers refer to the number of patent families

Patent applications are not published until after 18 months, so information after 2014 is not presented in Fig. 1. Patents expire after 20 years. Legal status is important for information on commercial exploitability of patents. Analysis of current legal status of the patent families of NTDs, presented in Fig. 2, reveals that almost 50% of the patents are non-active. This fact suggests that investing in NTDs has a low commercial value. Among the 17 NTDs identified, the prevalence of non-active patents is noticeably high in leprosy, schistosomiasis, trachoma and trematodiasis (see Additional file 2).

**Fig. 3:**
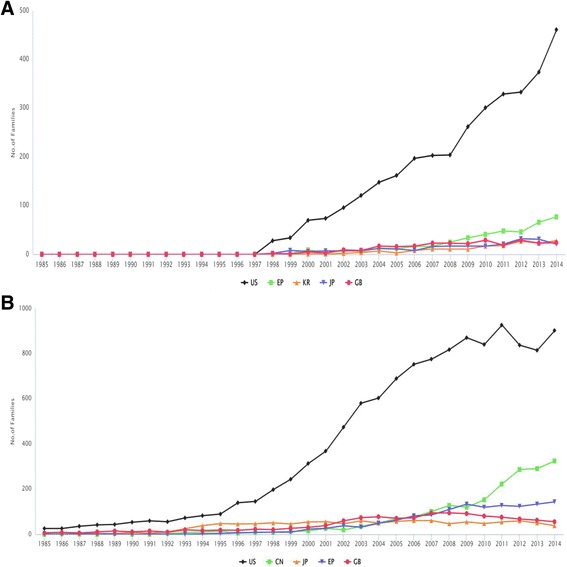
Number of granted patent families (**a**) and the total patent families (**b**) for the top five priority countries, by years. **a**: Main countries with source of inventions are the United States and the European Union-European Patent Office. Record numbers refer to the number of granted patent families. Priority countries are: US (United States), EP (European Union-European Patent Office), KR (Korea), JP (Japan), GB (Great Britain). **b**: Main countries with source of inventions are the United States and China. Record numbers refer to the number of patent families. Priority countries are: US (United States), CN (China), JP (Japan), EP (European Union-European Patent Office), GB (Great Britain)

Analyzing the top priority countries (countries where initial patent filing was submitted) for the granted patent families, it was observed that the main priority countries are the United States (US), European Union (EP), Korea (KR), Japan (JP) and Great Britian (GB) in the last 30 years. However, by focusing on the trend of total number of patent families, the leading countries are the US, China (CN), JP, EP, and GB. The gap between the first two priority countries is high, the US and China are with 6154 and 2423 patent families respectively. However, different patenting activity level of US and China can be detected by ratio of applications for patent families to granted patent families: 1898/3302 and 87/1525 respectively. With respect to NTDs, China appears as an emerging priority country compared with the US since 2010 as presented in Fig. 3. This trend is observed particularly for echinococcosis, rabies, schistosomiasis, and soil-transmitted helminthes. For example, China has a set priority for the soil-transmitted helminthiasis since 2010. Nonetheless, US has kept its leading role in intensive research on NTDs, such as leprosy, leishmaniasis and dengue. An interesting exception is observed for trematodiasis, which has Russia as its priority country.

**Fig. 4:**
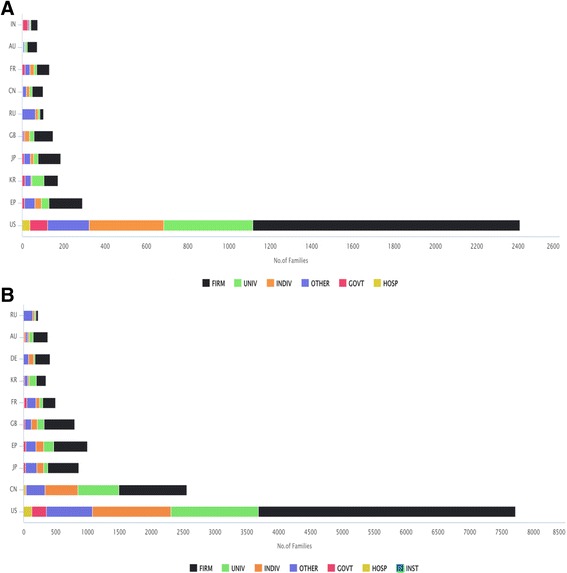
Number of granted patent families (**a**) and the total patent families (**b**) for the top ten priority countries by types of assignee. **a**: Firm (firms), indiv (individuals), univ. (universities), inst (non-profit institutions), govt (governments) and hosp (hospitals) are assignee types. “Others” classify the assignee names or company names which do not fall under these categories (university, government, non-profit institution, hospital, individuals). Record numbers referring to the number of granted patent families. Priority countries are: US (United States), EP (European Union-European Patent Office), KR (Korea), JP (Japan), GB (Great Britain), RU (Russia), CN (China), FR (France), AU (Australia), IN (India). **b**: Firm (firms), indiv (individuals), univ. (universities), inst (non-profit institutions), govt (governments) and hosp (hospitals) are assignee types. “Others” classify all the assignee names or company names which do not fall under these categories (university, government, non-profit institution, hospital, individuals). Record numbers refer to the number of patent families. Priority countries are: US (United States), CN (China), JP (Japan), EP (European Union-European Patent Office), GB (Great Britain), FR (France), KR (Korea), DE (Germany), AU (Australia), RU (Russia)

In the US, firms hold a large percentage of patent families in comparison to other interest groups such as individuals, universities, governments, and institutes. In China, France, Korea, and Russia, more than 50% of patents and applications were assigned to entities other than firms. By focusing on the assignee types of granted patent families, the role of firms is dominant, except for France, Korea and Russia. In Korea, the universities, and in Russia, no specified assignees are the major patent holders. Distribution of assignee types among priority countries is assessed in Fig. 4.

**Fig. 5:**
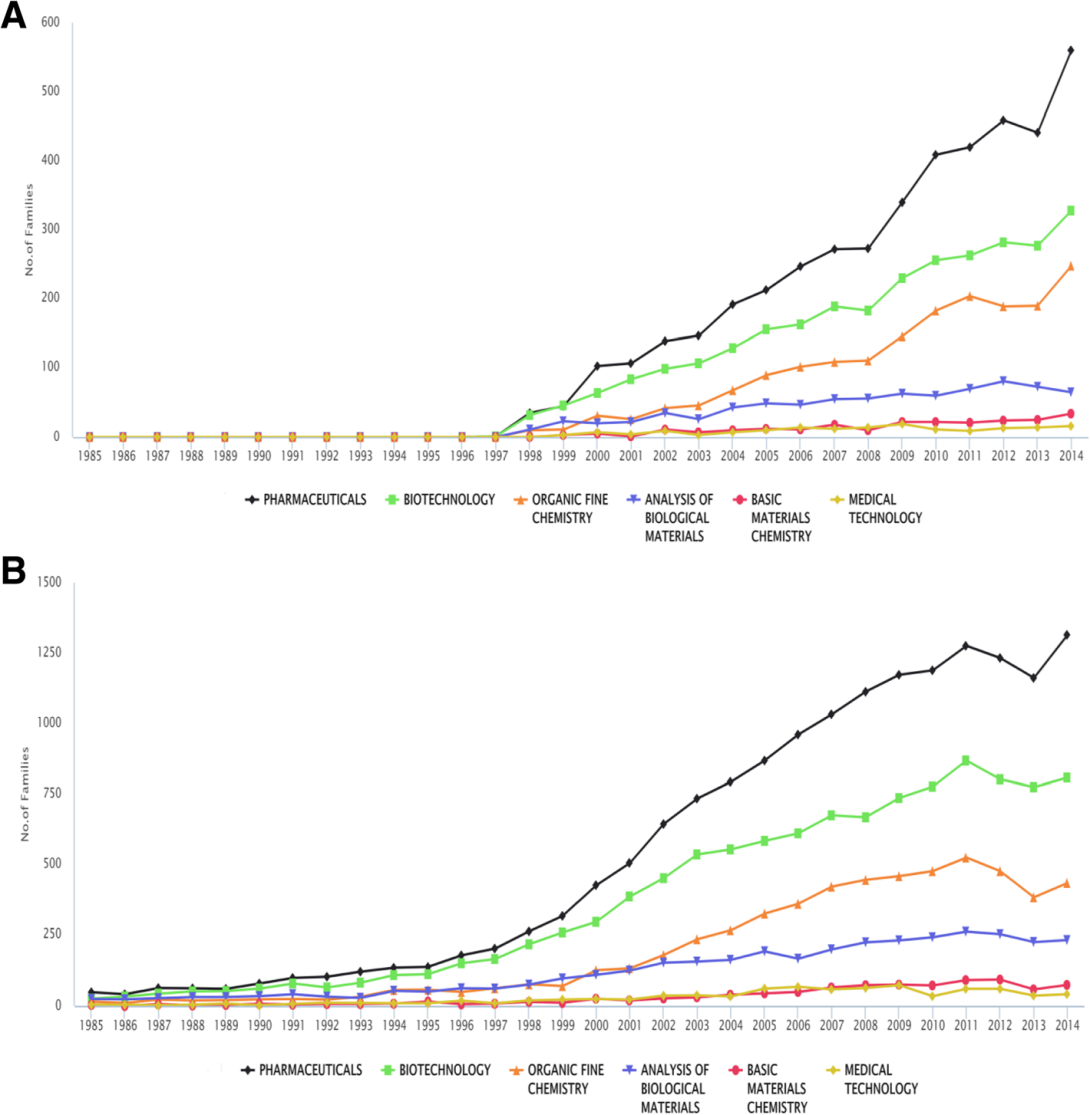
Number of granted patent families (**a**) and the total patent families (**b**) for the technological subdomains over time. **a**: The main technological subdomains are pharmaceuticals, biotechnology, organic fine chemistry, analysis of biological materials, basic materials chemistry and medical technology. Contininous growth can be observed especially in the field of pharmaceuticals, biotechnology, organic fine chemistry. Record numbers refer to the number of granted patent families. **b**: The main technological subdomains are pharmaceuticals, biotechnology, organic fine chemistry, analysis of biological materials, basic materials chemistry and medical technology. Contininous growth can be observed especially in the field of pharmaceuticals, biotechnology, organic fine chemistry between 1985 and 2011 followed by stagnations/slight decline. Record numbers referring to the number of patent families

Figure 5 provides an overview of the identified NTDs patent landscape in the form of technological fields. The main technological subdomains are pharmaceuticals, biotechnology, organic fine chemistry, analysis of biological materials, basic materials chemistry and medical technology. According to the NTDs trends, pharmaceuticals and biotechnology accounted for most patent families filed in the last 30 years. These two fields have shown substantial growth since 1985. Filings in organic fine chemistry have dropped in the last 10 years. The analysis of biological materials seems to be a popular field of innovation. Patent families for basic materials chemistry and medical technology have also shown substantial growth, in the overall analysis, but they account for a small portion of the filings. Focusing on the granted patent families, the stagnation/decline of the pharmaceuticals, biotechnology, organic fine chemistry fields are not yet present. The percentage of technical subdomains (pharmaceuticals, biotechnology, organic fine chemistry, analysis of biological materials, basic materials chemistry and medical technology) for alive versus non-alive patent families were similar. The highest proportions were observed in the pharmaceutical field, and the high proportion of dead patent families in the pharmaceutical is as a result of a decline in patent applications. Additionally, by comparing the technical subdomains of the IDM and PCT groups examined, it was observed that they have the same subdomains ranking order. However, while the hierarchy among subdomains of IDM is rather constant, there are changes in the positions of the subdomains of PCT. A very important observation is the clear decline in the number of patent families for pharmaceuticals and organic fine chemistry in the group of PCT.

The IPC classification of NTDs patents showed that class A61 is the most prominent class in which NTDs research patents are being categorised. In respect of this categorisation, A61K39/00 (medicinal preparations containing antigens or antibodies) is the most dominant IPC subgroup within the A61 class. Detailed research focus of each disease is presented by IPC subgroups in Additional file 2.

**Fig. 6:**
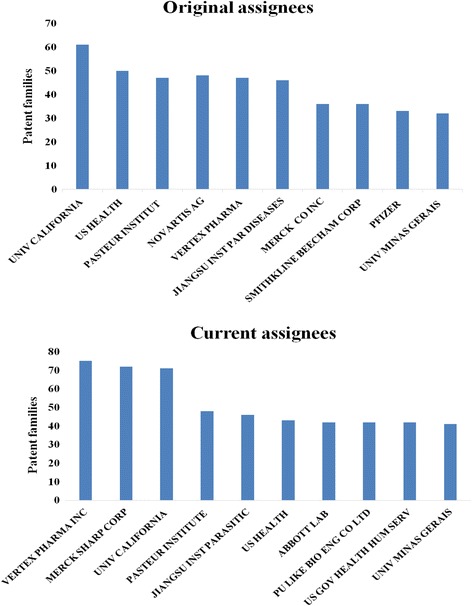
Original and current patent assignees. For patent families of all NTDs, University of California and US Health are the major original assignees, and Vertex Pharmaceuticals, Merck Sharp Incorporation are the main current assigness. Record numbers refer to the number of patent families

For a patent landscape analysis, analyzing the distribution of active patent applicants in a research field is important. With respect to NTDs research, a lack of dominant assignees (more than 33% of patents) was observed (Fig. 6). The main original assignees for NTDs research are governmental institutions and universities, such as Univeristy of California (US) or Pasteur Institute (FR). Among current assignees, firms such as Merck, Vertex Pharma Inc. tend to take more financial risks on NTDs research.

## Discussion

Long term trends reveal a continuous growth in the number of patent families of NTDs with a slight decrease after 2008. This continuous growth in trends is not uniform for all the NTDs. For example, there has been a significant decline in trachoma and leprosy research. Focusing on the granted patent families, a stagnation can be observed after 2008, not a decline. Additionally, previously marginalized diseases such as dracunculiasis were successful in attracting research interest in the last 10 years. However, global patenting trend is in sharp contrast with the findings on NTDs in this study. In the last 20 years, the total number of global patenting applications has tripled [23], but patent application increase on NTDs has not yet reached this rate. In order to better demonstrate the proportions of patenting activity, the number of patent families, corrected for normalized DALY (2015), was compared with a few other selected similarly robust social-, health- and economic impact diseases such as HIV/AIDS, malaria, cardiovascular diseases, cancers, and lung cancer [14]. The gap between patenting NTDs and cardiovascular diseases/cancers is striking; the number of filed patents for cardiovascular diseases or cancer is at least 200 times larger than NTDs. Individual NTDs lag behind lung cancer, malaria or HIV/AIDS in patenting activities. Background data is presented in Additional file 3. R&D interests among NTDs is very uneven. Leishmaniasis, dengue, schistosomiasis and rabies accounted for most of the growth in patenting activities. An obvious link between disease burden or availability of treatment (eg. PCT or IDM category) and patenting activity could not be identified in this study. This study finding shows that there is a limited attractiveness in this field, and this is consistent with previous articles on novel drug and vaccine landscape of NTDs by showing decrease as a tendency. Cohen et al., found 32 new chemical entities between 1975 and 1999, while between 2000 and 2009, there was only 26 newly approved drugs and vaccines for NTDs [24]. Pedrique et al., reported that most progress towards reducing the burden of NTDs focus on repurposing or reformulating existing drugs [25]. The Bill and Melinda Gates Foundation which has funded Policy Cures Research to conduct the last nine annual G-FINDER surveys also found stagnation in terms of new chemical entities of NTDs [26].

The analysis of this study also showed that the US is losing its position as a major priority country. This is consistent with the fact that China now drives global patent applications beginning with a new record achieved in 2015 [22]. Diversity between original and current assignees such as US Health vs Merck & Co.; Pasteur vs Vertex Pharma Institute have been found in the patent database. This is a clear sign of emerging new interested parties. However, a high number of non-firm assignees indicates the limited level of industrial maturity in this field. A higher percentage of firms are assignees resident in the US in the field of NTDs compared to China. However, in China, there is a high proportion of patent families linked to univeristies or individuals which indicates high research activity.

An additional concern could be the high proportion of expired NTDs patents. Expired patents have limited strategic value to their assignees. This is because others cannot be excluded from using the invention(s) disclosed in the patent. However, information from expired patents may be relevant in the mitigation of NTDs, and can be used by non-governmental organizations (NGOs) or private-public partnerships who are key players to curbing the spread of NTDs [27].

The overall description of information contained in patent families was through technology fields. The main technology subdomains with emerging trends are pharmaceuticals and biotechnology. Many of the patents retrieved have strong focus towards medicinal preparations containing antigens or antibodies.

Based on the method of patent landscape analysis, patent families of each NTD were identified, merged and analysed to get overall insights regarding the trends, topics, and stakeholders in this field. This work could be a robust basis for future research in order to plan, monitor or justify decisions for R&D policies.

Although, this paper argues that pursuing R&D efforts in NTDs through the development of new innovations is important. R&D does not provide answers for several observed problems within the NTDs. It is important to pay attention to the broad social factors affecting NTDs; parallel improvements in hygiene, sanitation and access to medical care cannot be overlooked. Finding effective ways for development seems possible through public-private partnerships or new innovative alliances, established on case by case basis. Ways of addressing social challenges of NTDs may be found by taking good examples from HIV/AIDS management [28].

It is important to note that there are a number of methodological limitations in this study. There are limitations to the use of patent data as an indicator of technological development. This is primarily because not all inventions meet patentability standards, and inventors tend to rely on secrecy or other appropriate means to protect their inventions. Although, the developed search criteria facilitated the retrieval of patents of each NTD, it limited the absolute scope of a patent search. This simply mean that some patents might have not been included in the dataset intentionally. This is, however, a general limitation of all patent landscape analyses. Additionally, there is usually a time lag of at least 18 months between the first patent filing and the patent publication; and even longer time is used for granting.

Finally, R&D analysis alone cannot show trends and future scencarios of research fields. Patent landscape analyses are quite simple, yet an effective way of planning and/or monitoring R&D of NTDs.

## Conclusion

The filing pattern of patent families reviewed strongly reveals limited efforts on research and development of NTDs, whereas it is crucial to intensify research efforts into NTDs. Involving new players, such as more NGOs may help to mitigate and reduce the burden of NTDs. In this work, patent landscape analysis has been presented as a reliable method that can be adopted for providing feedback on overall research progress of identified NTDs. R&D incentives are not sufficient to solve the problem of inaccessibility of essential medicines in regions affected by NTDs. Strengthening the health systems, political and global health efforts will be of immense benefits to the most affected regions.

## Additional files

Additional file 1: Final search terms of NTD. (PDF 23 kb)

Additional file 2: Detailed analyis of NTDs. (PDF 2490 kb)

Additional file 3: An inter-disease comparison of patenting activity. (PDF 20 kb)

## Abbreviation

IDM: Innovative and Intensified Disease Management
IPC: International Patent Classification
MDA: Mass Drug Administraion
NGOs: Non-Governmental Organizations
NTDs: Neglected Tropical Diseases
PCT: Preventive Chemotherapy and Transmission Control
R&D: Research and Development
WHO: World Health Organization
