# Supplementary material for: Patent landscape of neglected tropical diseases: an analysis of worldwide patent families
Source: Global Health. 2017 Nov 14;13:82. doi: 10.1186/s12992-017-0306-9 (PMC5686799; doi:10.1186/s12992-017-0306-9)
Supplement: Supplementary file 1 — Final search terms of NTD. (PDF 23 kb) [file 12992_2017_306_MOESM1_ESM.pdf]

Additional File 1: Final search terms of NTD

Disease	Search Terms
**Buruli ulcer**	(Mycobacterium ulcerans) OR (M. ulcerans) OR (Buruli Ulcer) OR (non-tubercul* mycobacter*)
**Chagas**	(Chagas disease) OR (trypanosomias*) OR (Trypanosoma cruzi) OR (T. cruzi)
**Dengue**	(Dengue w5 virus*) OR (Dengue disease*) OR (Dengue w5 fever) OR (Breakbone fever) OR (Chikungunya w5 fever) OR (Chikungunya w5 virus*)
**Dracunculiasis**	(Dracunculiasis) OR (Guinea Worm) OR (Guinea-worm disease) OR (Dracunculus medinensis)
**Echinococcosis**	(Echinococcos*) OR (hydatid disease) OR (hydatidosis) OR (echinococcal disease) OR Echinococcus OR (cystic echinococcosis) OR (alveolar echinococcosis) OR (polycystic echinococcosis) OR (unicystic echinococcosis)
**Food-borne Trematodiasis**	(Food-borne Trematodias*) OR (Clonorchiasis OR Opisthorchiasis OR Fascioliasis OR Paragonimiasis) OR (Chinese liver fluke) OR (Clonorchis sinensis) OR (Opisthorchis viverrini) OR (Opisthorchis felineus) OR (fascioliasis OR fasciolasis OR distomatosis) OR (liver rot) OR (Fasciola hepatica) OR (Fasciola gigantica) OR (Paragonimus westermani) NOT (ruminant OR animals OR cattle OR Schistosomiasis OR schistosoma)
**Human African Trypanosomiasis**	(African Trypanosomiasis*) OR (sleeping sickness) OR (nagana) OR (African Trypanosomiasis*) OR (Trypanosoma brucei gambiense) OR (Trypanosoma brucei rhodesiense) NOT (Chagas OR Plasmodium)
**Leishmaniasis**	Leishmanias* OR leishmania OR leishmaniosis
**Leprosy**	(Leprosy) OR (Hansen disease) OR (Mycobacterium leprae) OR (Mycobacterium lepromatosis) OR (nontubercul* mycobacter*) NOT (Mycobacterium ulcerans) NOT (Mycobacterium tuberculos*)
**Lymphatic Filariasis**	(WUCHERERIA BANCROFTI) OR (BRUGIA MALAYI) OR (lymphatic filariasis) OR (Elephantias*) OR (Brugia timori)
**Onchocerciasis**	Onchocercias* OR (Onchocerca volvulus) OR (river w5 blindness) NOT (trachoma)
**Rabies**	(rabies w5 virus) OR (lyssa*) OR (rabies w5 infection*) OR (rabies w5 disease)

**Schistosomiasis**	(Schistosomiasis) OR (snail fever) OR (bilharzia) OR (Katayama fever) OR (Schistosomiasis haematobia) OR (Schistosomiasis japonica) OR (Schistosomiasis mansoni) OR (Schistosoma) OR (blood-flukes)
**Soil-transmitted helminthiasis**	(Soil transmitted w5 helminthias*) OR (soil transmitted w5 helminths) OR (ascarias*) OR (hookworm infection*) OR (hookworm disease*) OR (ancylostomias*) OR (necatorias*) OR (whipworm infection*) OR (Ascaris lumbricoides) OR (Necator americanus) OR (Ancyclostoma duodenale) OR (Trichuris trichiura) OR (Trichurias*)
**Taeniasis**	(taeniasis) OR (Taenia solium) OR (pork tapeworm) OR (Taenia saginata) OR (beef tapeworm) OR (Taenia asiatica) OR (Taenia w5 Infection*)
**Trachoma**	(Trachoma) OR (Egyptian Ophthalmia) OR (Chlamydia trachomatis) OR (granular conjunctivit*)
**Yaws**	(Treponema pallidum) OR (Yaws disease*) OR (Thymosis) OR (framboesia) OR (Frambesia) OR (Treponema pertenue)

Keywords and terms were searched in any of the Title, Abstract or Claims fields. W5 symbol: two words that may appear side by side or separated by up to five words. * Wildcard: symbol that broadens a search by finding words that start with the same letters.

Search terms of selected diseases

**Cardiovascular disease**	(Arrhythmias OR Cardiac OR (Carcinoid Heart Disease) OR (Cardiac Output) OR (High Cardiac Output) OR (Low Cardiac) OR Tamponade OR Cardiomegaly OR Cardiomyopathies OR Endocarditis OR (Heart Aneurysm) OR (Heart Arrest) OR (Heart Defects) OR (Congenital Heart) OR (Heart Rupture) OR (Heart Valve Diseases) OR (Myocardial Ischemia) OR (Myocardial Stunning) OR (Pericardial Effusion) OR Pericarditis OR Pneumopericardium OR (Postpericardiotomy Syndrome) OR (Pulmonary Heart Disease) OR (Rheumatic Heart Disease) OR (Ventricular Dysfunction) OR (Ventricular Outflow Obstruction)) OR (cariovascular OR (heart disease) OR angina OR (heart failure) OR (heart attack)) OR (Aneurysm OR Angiodysplasia OR Angioedema OR Angiomatosis OR (Aortic Diseases) OR (Arterial Occlusive Diseases) OR (Arteriovenous Malformations) OR (Capillary Leak Syndrome) OR (Cerebrovascular Disorders) OR (Ischemic Compartment Syndrome) OR (Diabetic Angiopathies) OR (Embolism) OR (Thrombosis) OR (Hand-Arm Vibration Syndrome) OR (Hemorrhoids) OR (Hemostatic Disorders) OR (Hepatic Veno-Occlusive Disease) OR Hyperemia OR Hypertension OR Hypotension OR (Mesenteric Ischemia) OR (Optic Neuropathy) OR (Ischemic Peripheral Vascular Disease) OR Prehypertension OR (Pulmonary Veno-Occlusive Disease) OR (Reperfusion Injury) OR (Retinal Vein Occlusion) OR (Scimitar Syndrome) OR (Spinal Cord Vascular Diseases) OR (Splenic Infarction) OR (Stenosis Pulmonary Vein) OR (Superior Vena Cava Syndrome) OR Telangiectasis OR (Thoracic Outlet Syndrome) OR Varicocele OR Varicose OR Veins OR (Vascular Fistula) OR (Vascular Neoplasms) OR (Vascular System Injuries) OR Vasculitis OR Vasoplegia OR (Venous Insufficiency)))
**Cancer**	(Cancer OR (Anti*cancer) OR Chemotherap* OR Oncol* OR Carcinog* OR Neoplas* OR Tumor OR Metastat* OR Malignan*)
**Lung cancer**	(pulmonary cancer) OR (lung cancer) OR (lung neoplasm) OR ((carcinoma OR cancer OR metastasis OR neoplasm OR tumor) AND (lung OR Trachea OR bronchus))
**HIV/AIDS**	(HIV OR (Human Immunodeficiency Virus)) OR (AIDS OR (acquired

	immune deficiency syndrome)
**Malaria**	Malaria OR plasmodium

Keywords and terms were searched in any of the Title, Abstract or Claims fields. W5 symbol: two words that may appear side by side or separated by up to five words. * Wildcard: symbol that broadens a search by finding words that start with the same letters.
