# Supplementary material for: Patent landscape of neglected tropical diseases: an analysis of worldwide patent families
Source: Global Health. 2017 Nov 14;13:82. doi: 10.1186/s12992-017-0306-9 (PMC5686799; doi:10.1186/s12992-017-0306-9)
Supplement: Supplementary file 2 — Detailed analyis of NTDs. (PDF 2490 kb) [file 12992_2017_306_MOESM2_ESM.pdf]

Buruli ulcer

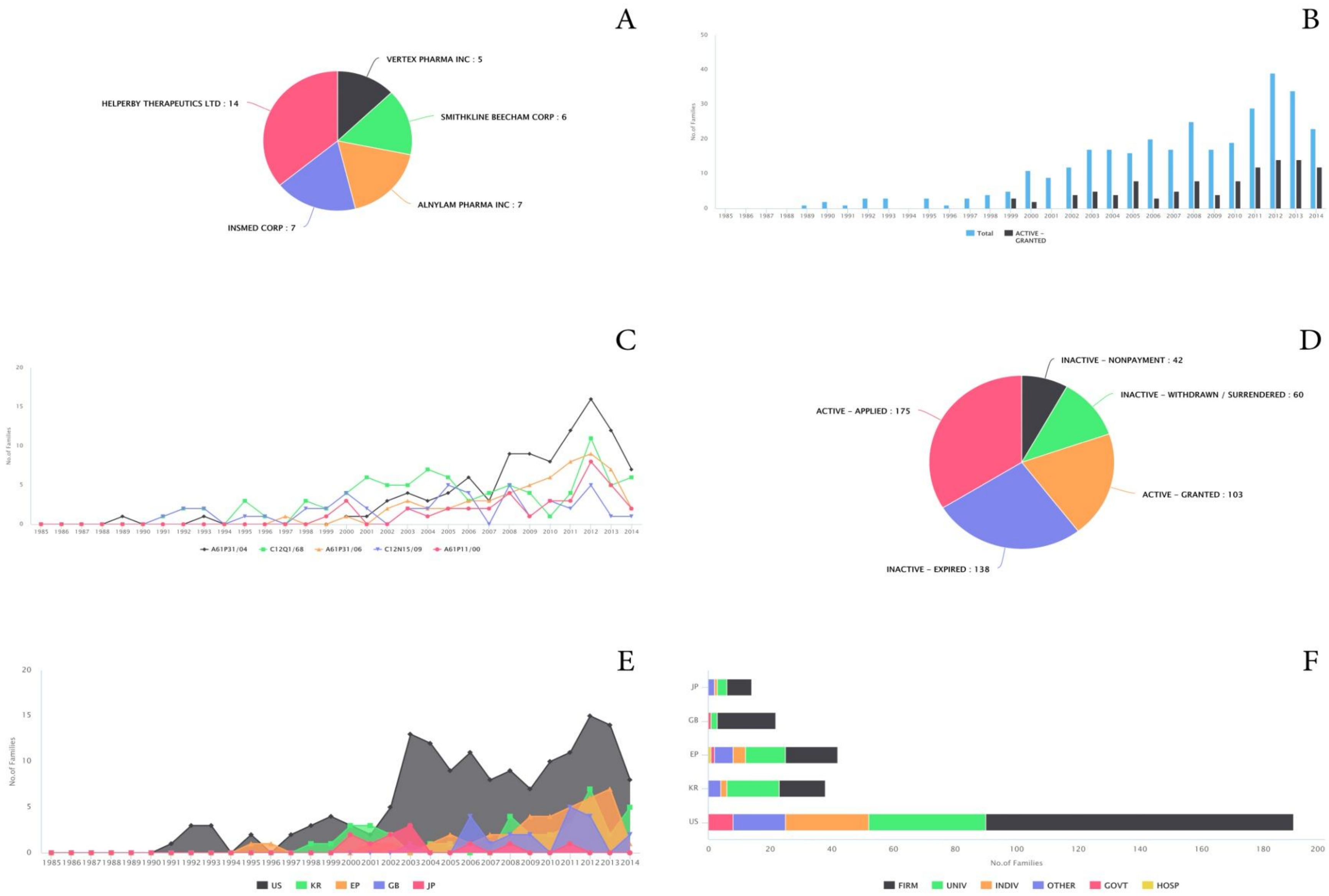

Chagas disease

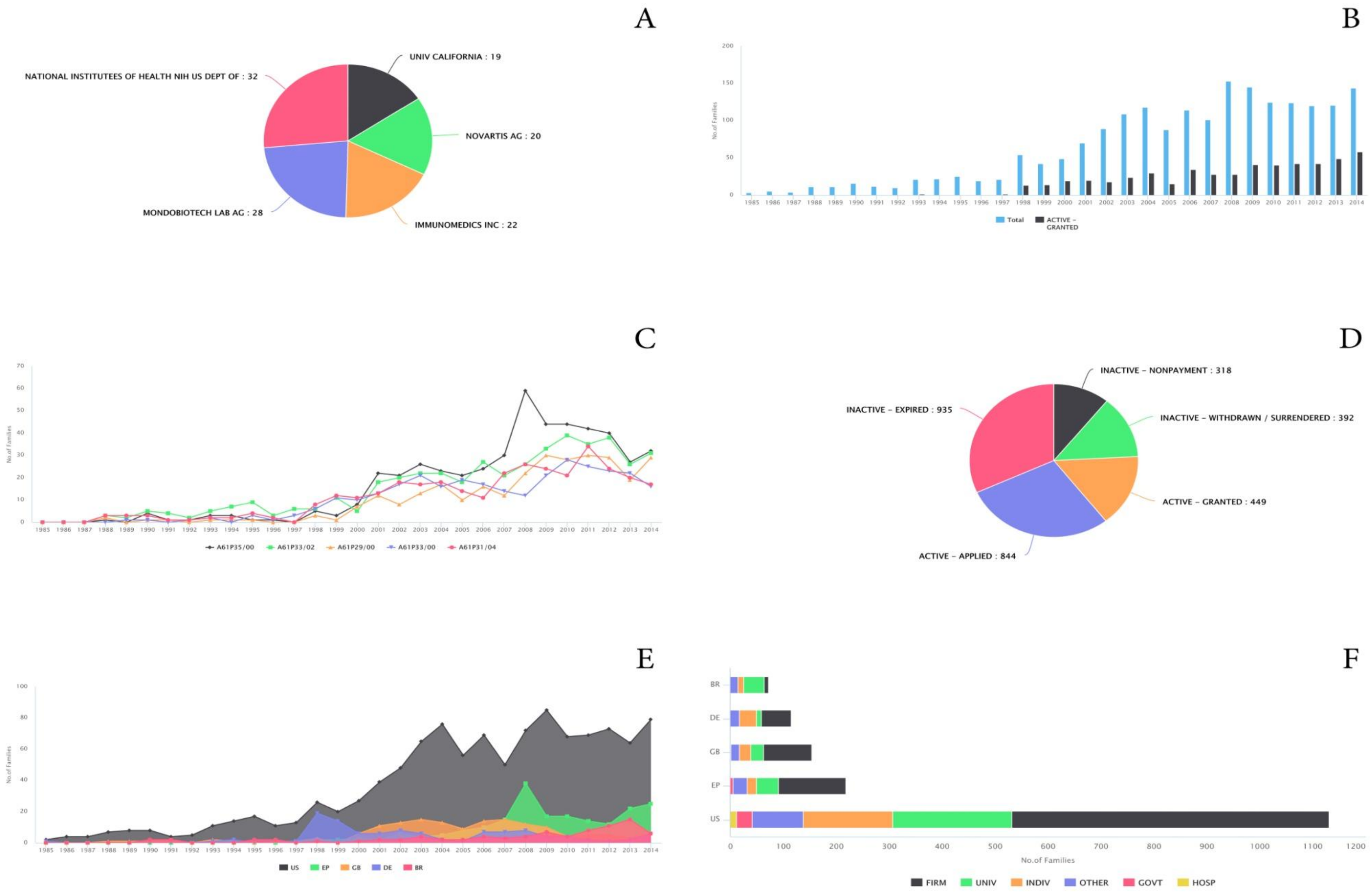

Dengue

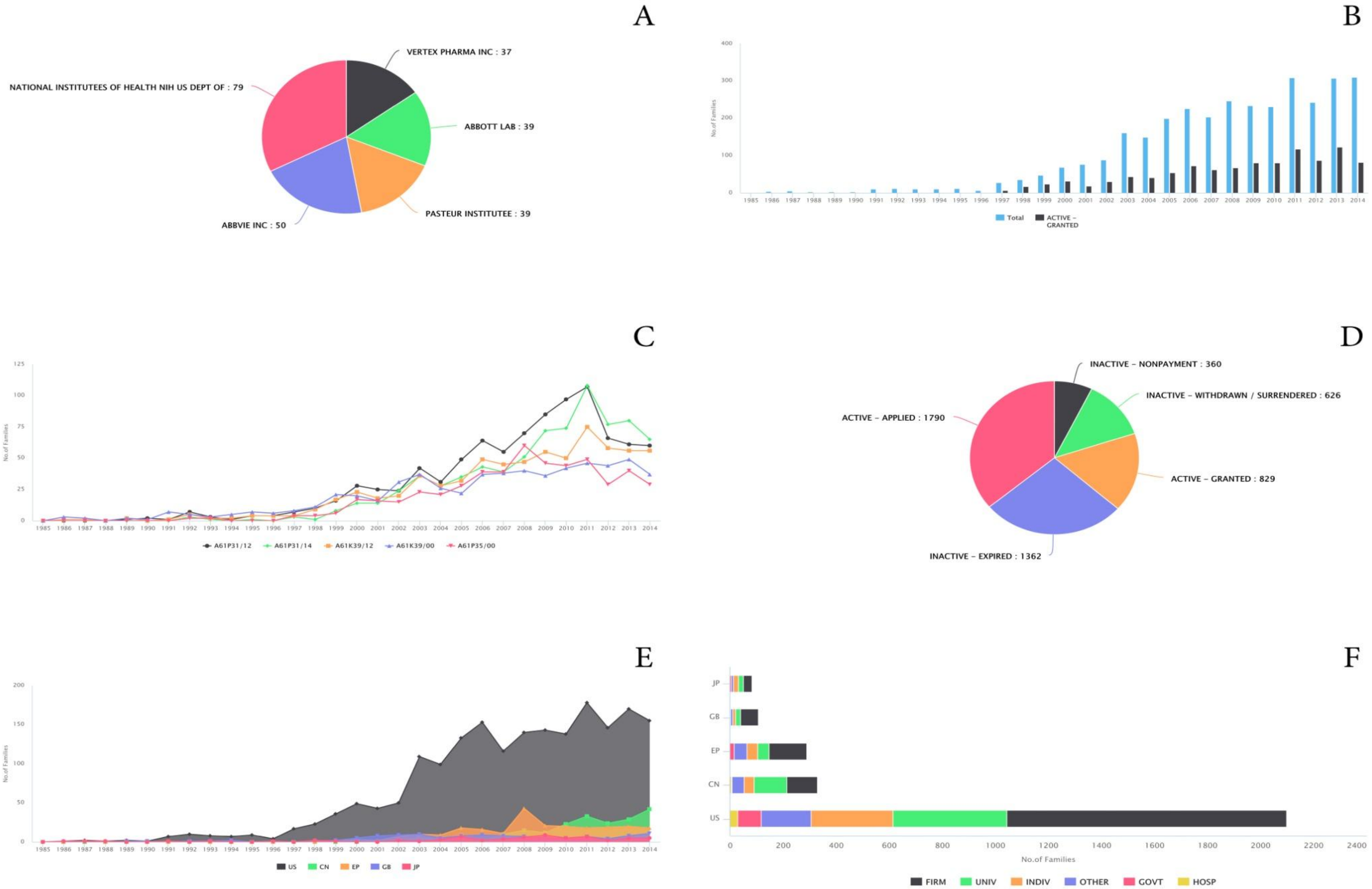
A
VERTEX PHARMA INC : 37
NATIONAL INSTITUTEES OF HEALTH NIH US DEPT OF : 79
ABBOTT LAB : 39
PASTEUR INSTITUTEE : 39
ABBVIE INC : 50
B
No.of Families
Total
ACTIVE - GRANTED
C
No.of Families
A61P31/12
A61P31/14
A61K39/12
A61K39/00
A61P35/00
D
INACTIVE - NONPAYMENT : 360
INACTIVE - WITHDRAWN / SURRENDERED : 626
ACTIVE - APPLIED : 1790
ACTIVE - GRANTED : 829
INACTIVE - EXPIRED : 1362
E
No.of Families
US
CN
EP
GB
JP
F
JP
GB
EP
CN
US
No.of Families
FIRM
UNIV
INDIV
OTHER
GOVT
HOSP

Dracunculiasis

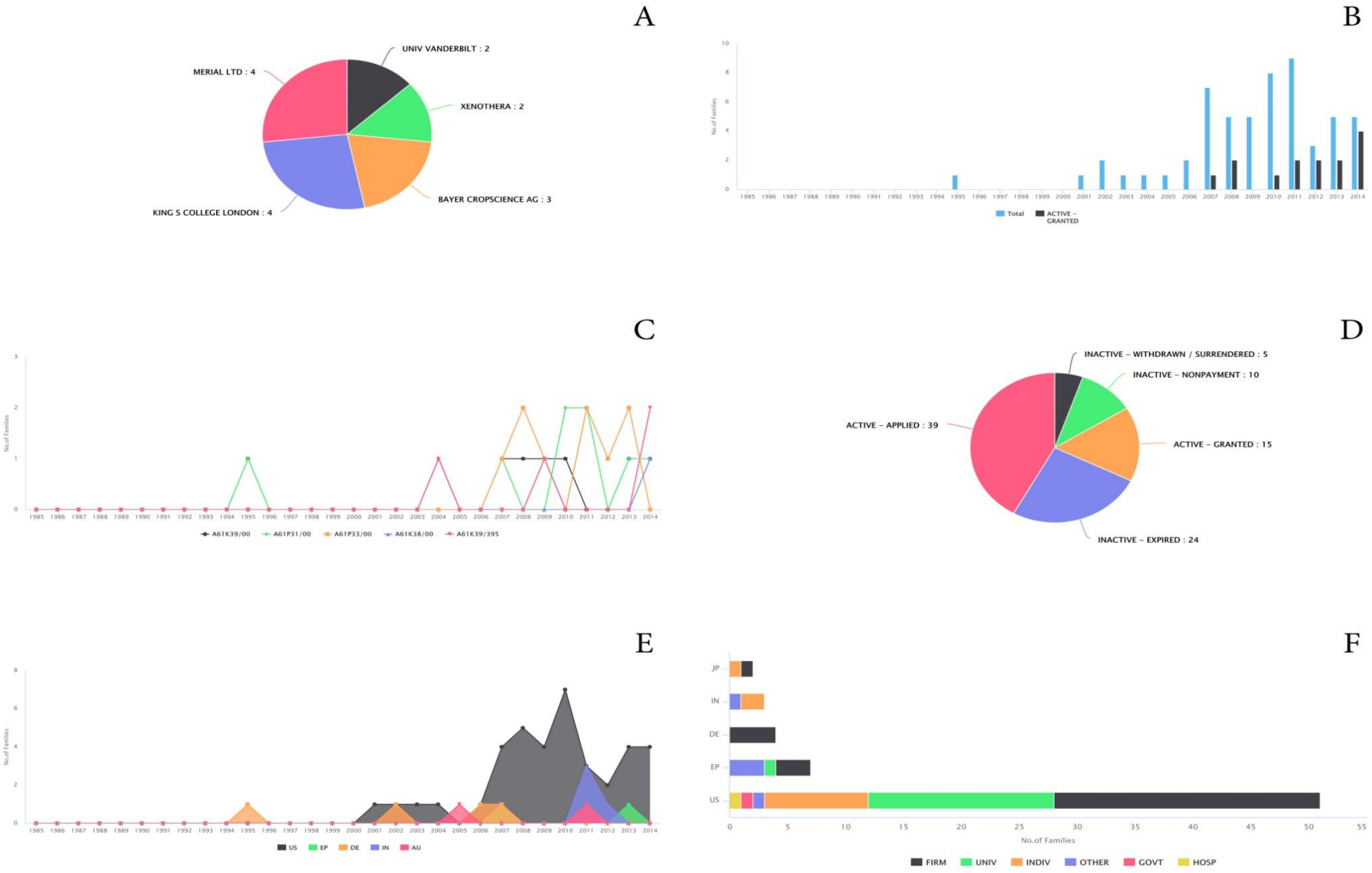

Echinococcosis

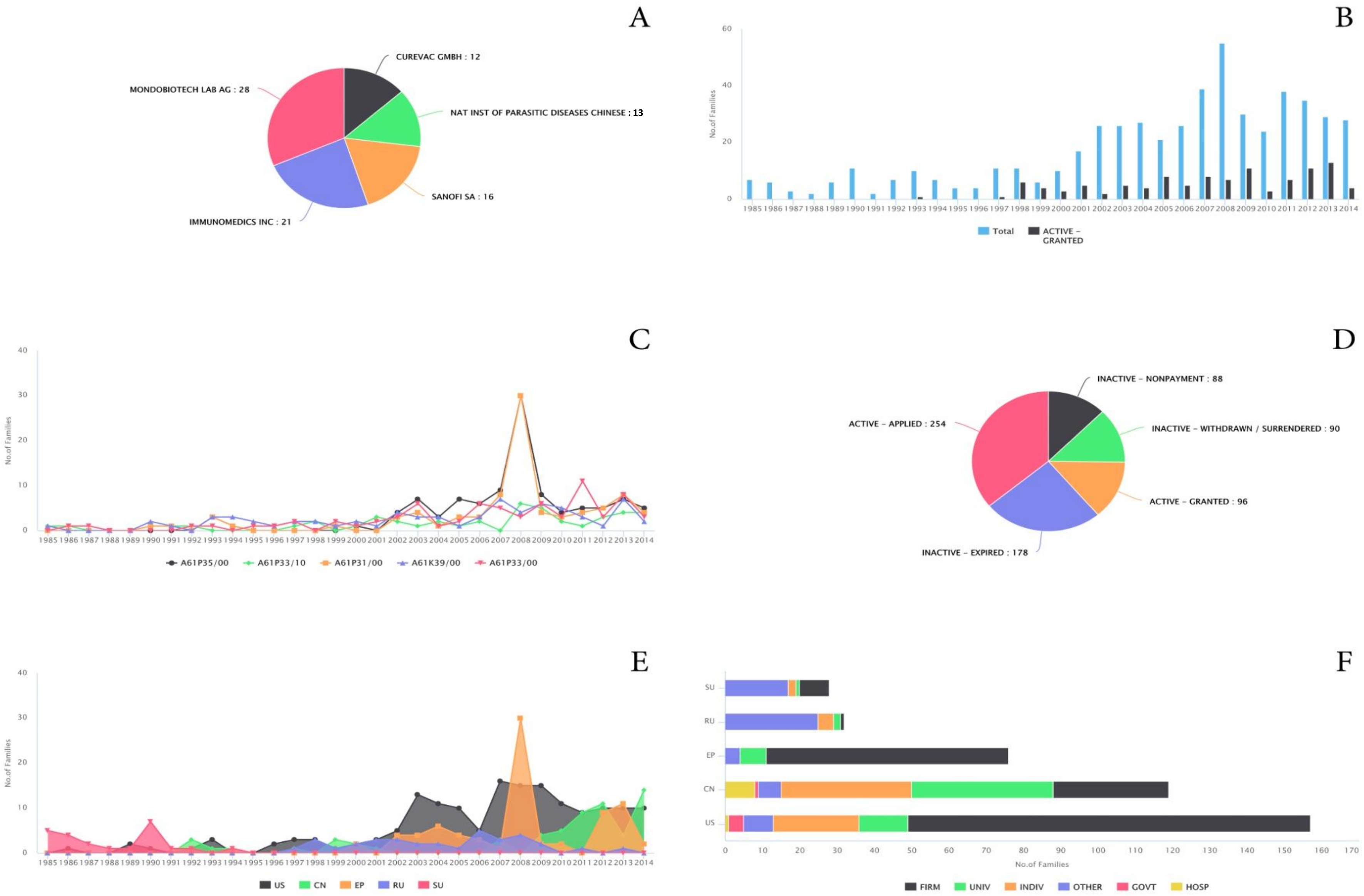

Food-borne trematodiasis

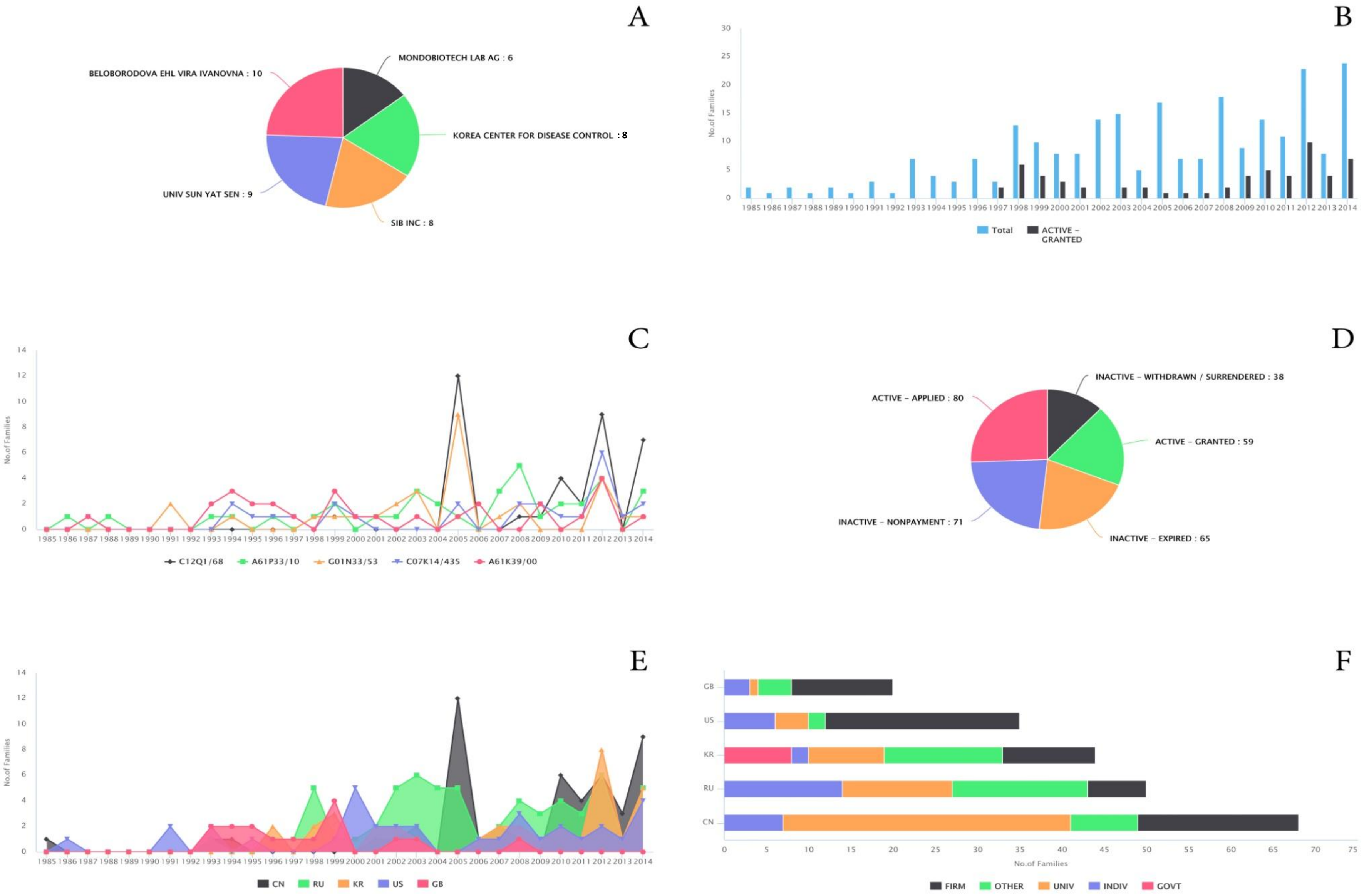

Human African trypanosomiasis

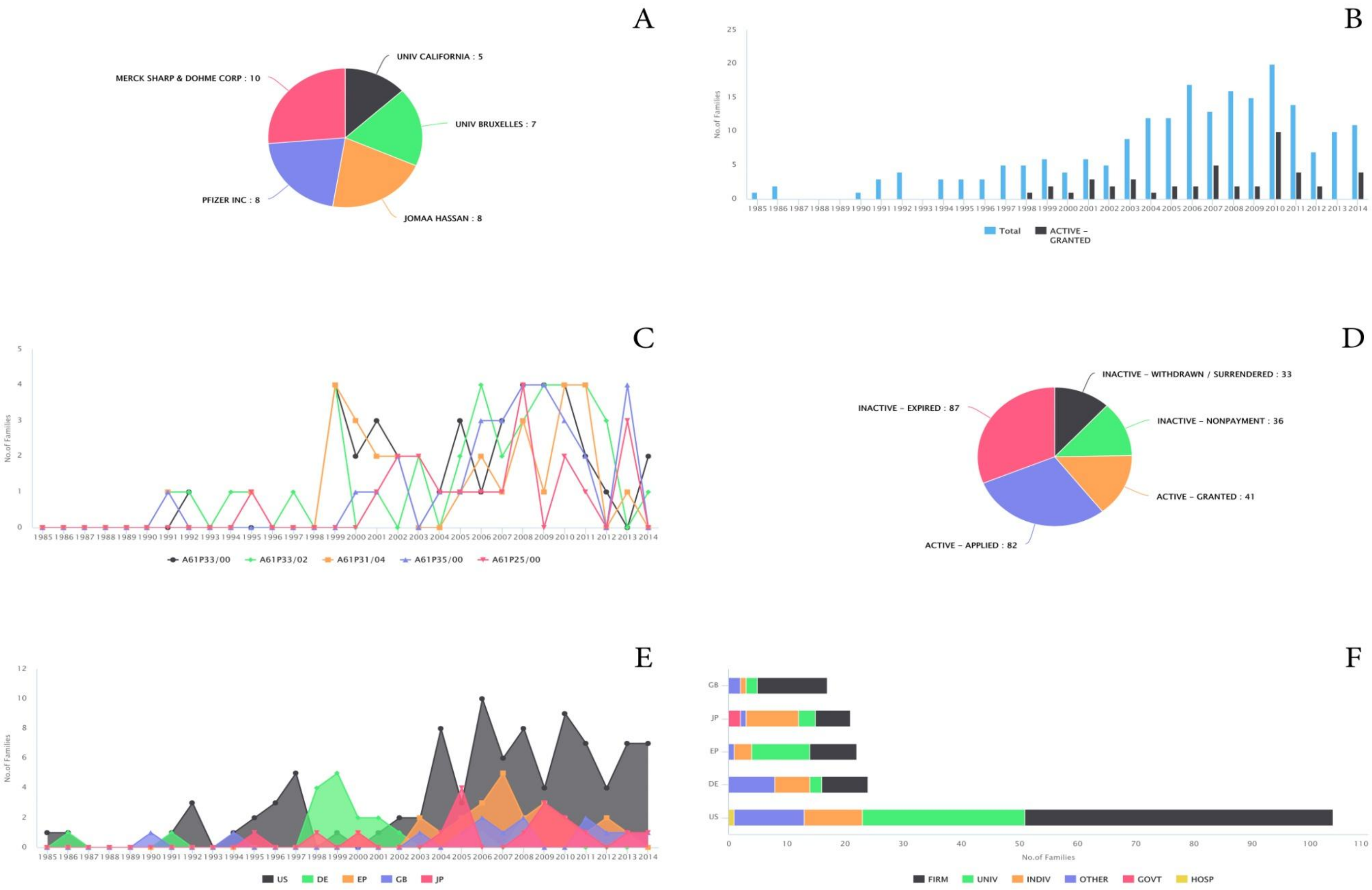

Leishmaniasis

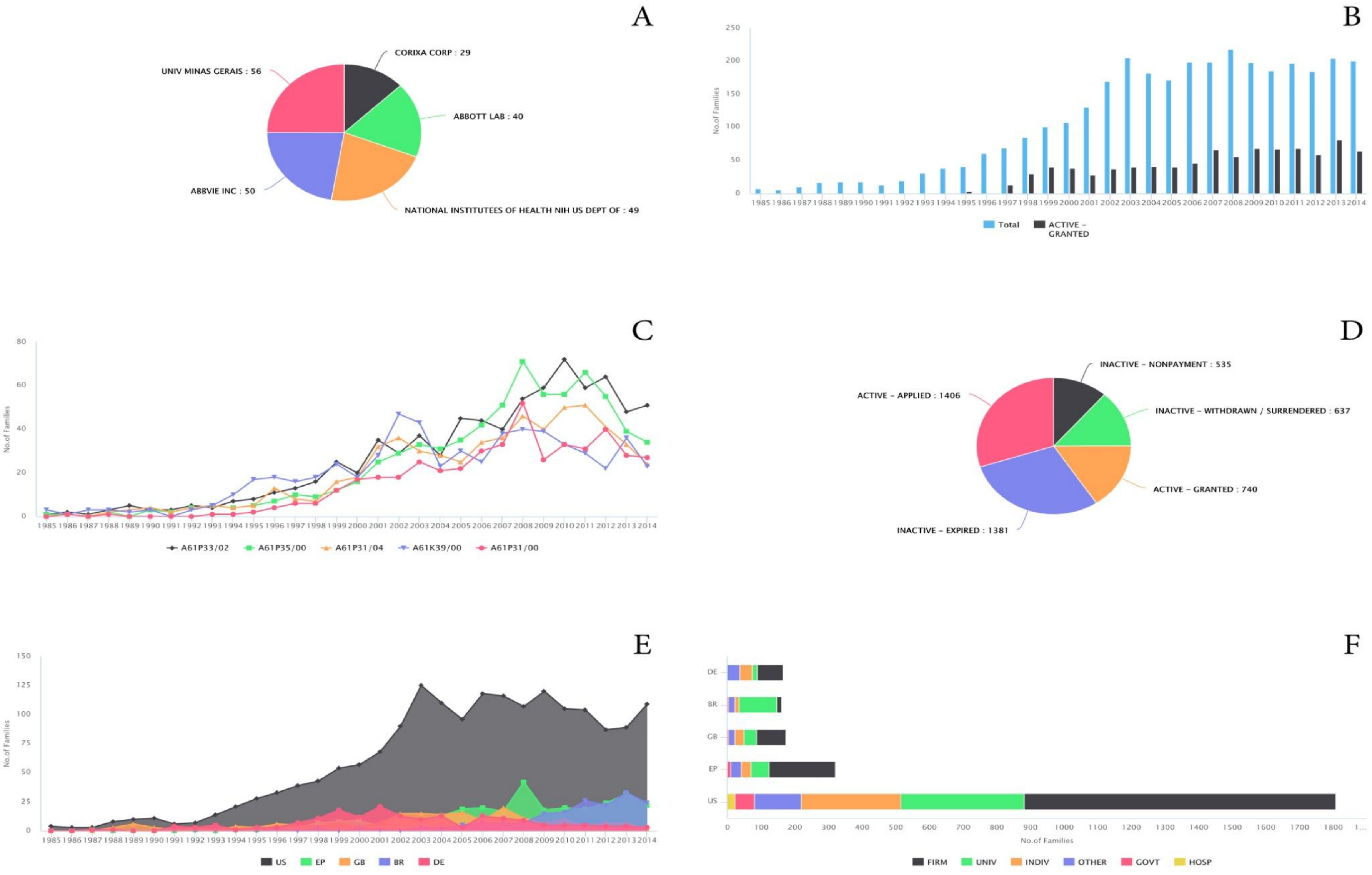

Leprosy

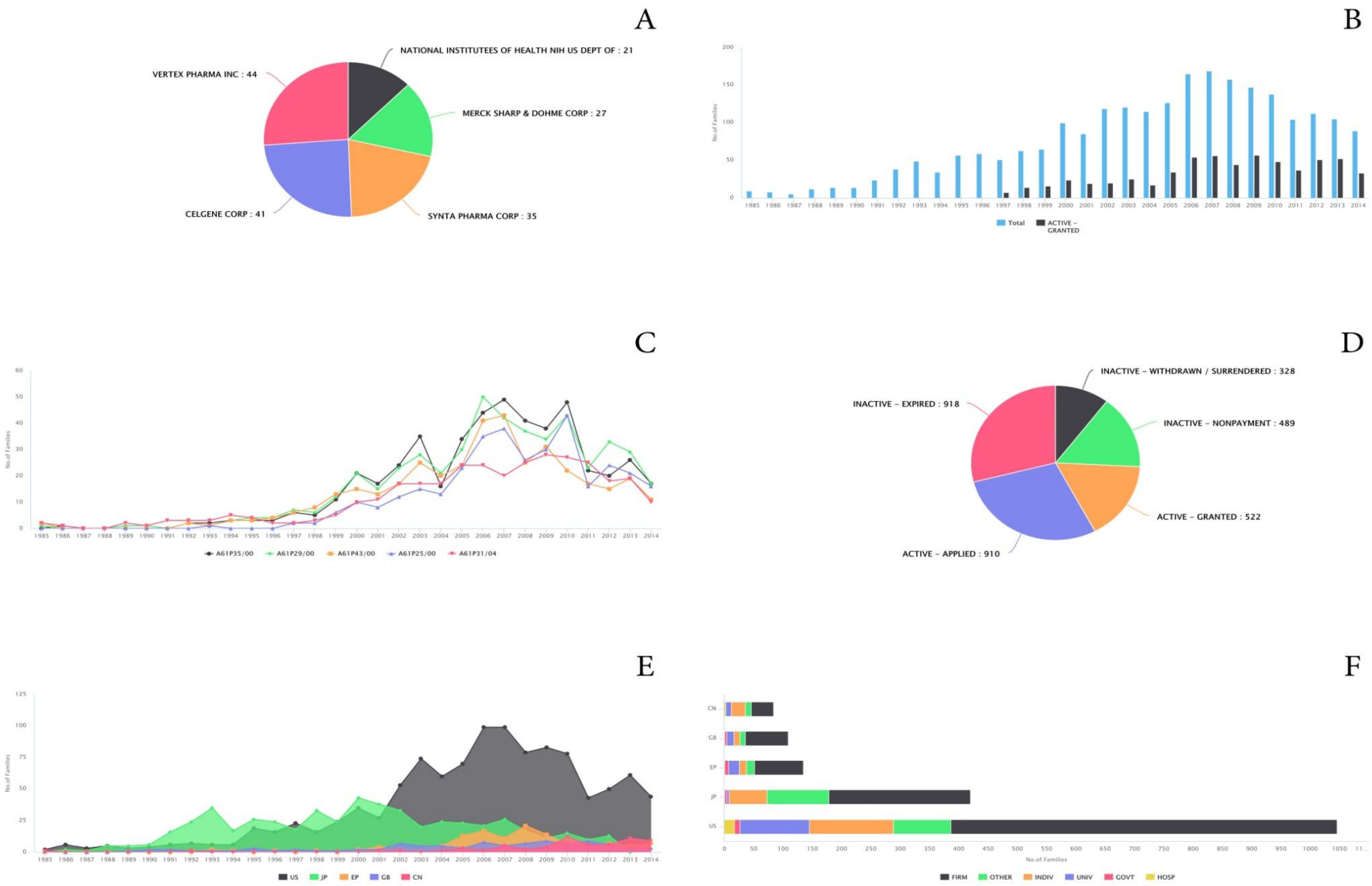
A
NATIONAL INSTITUTEES OF HEALTH NIH US DEPT OF : 21
VERTEX PHARMA INC : 44
MERCK SHARP & DOHME CORP : 27
CELGENE CORP : 41
SYNTA PHARMA CORP : 35
B
No.of Families
Total
ACTIVE – GRANTED
C
No.of Families
A61P35/00
A61P29/00
A61P43/00
A61P25/00
A61P31/04
D
INACTIVE – WITHDRAWN / SURRENDERED : 328
INACTIVE – EXPIRED : 918
INACTIVE – NONPAYMENT : 489
ACTIVE – GRANTED : 522
ACTIVE – APPLIED : 910
E
No.of Families
US
JP
EP
GB
CN
F
CN
GB
EP
JP
US
No.of Families
FIRM
OTHER
INDIV
UNIV
GOVT
HOSP

Lymphatic filariasis

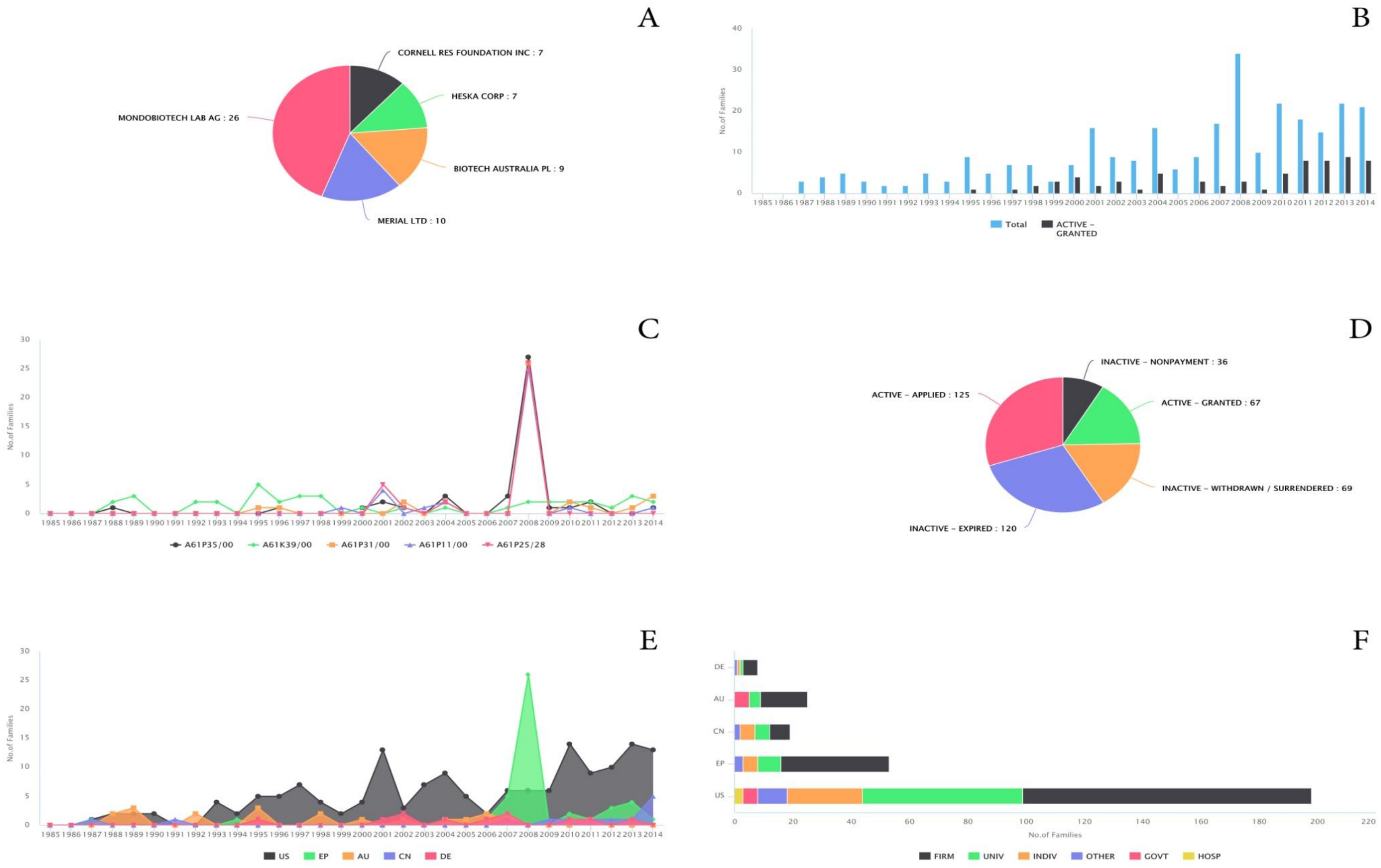

Onchocerciasis

A

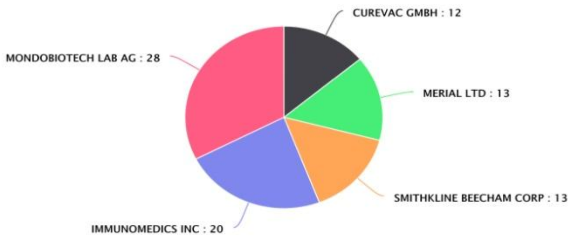

B

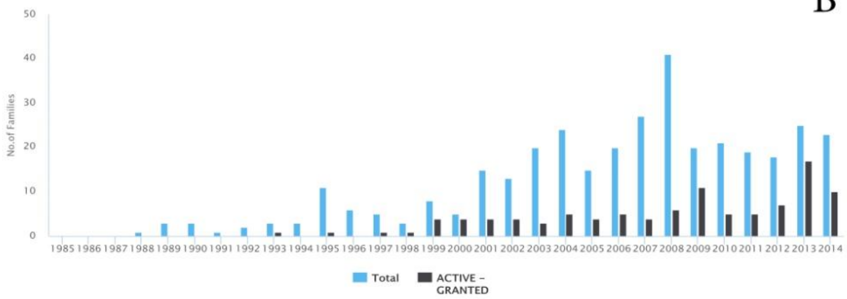

C

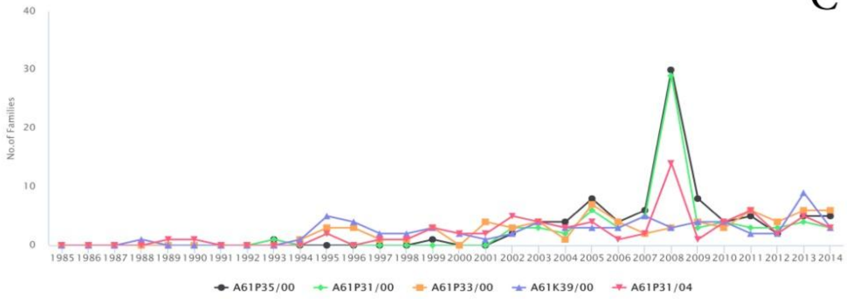

D

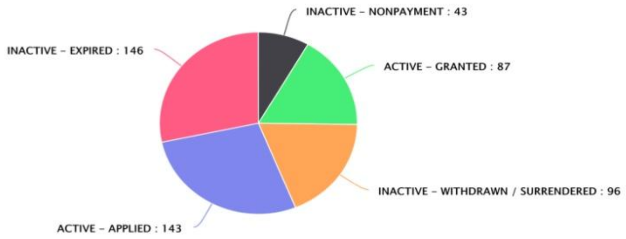

E

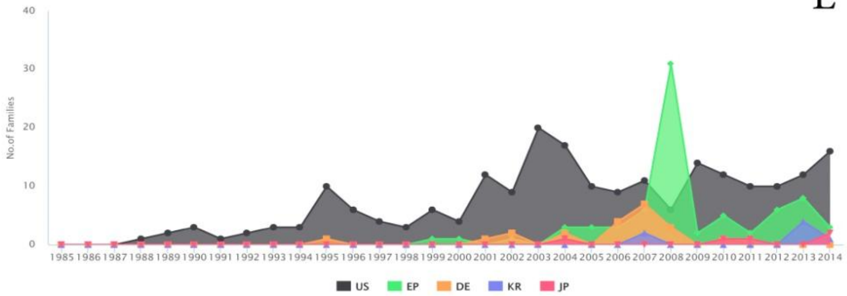

F

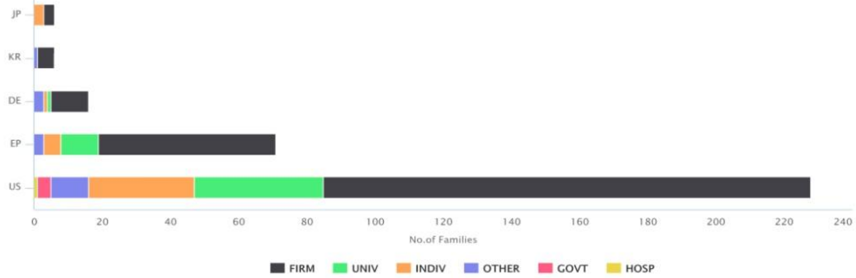

Rabies

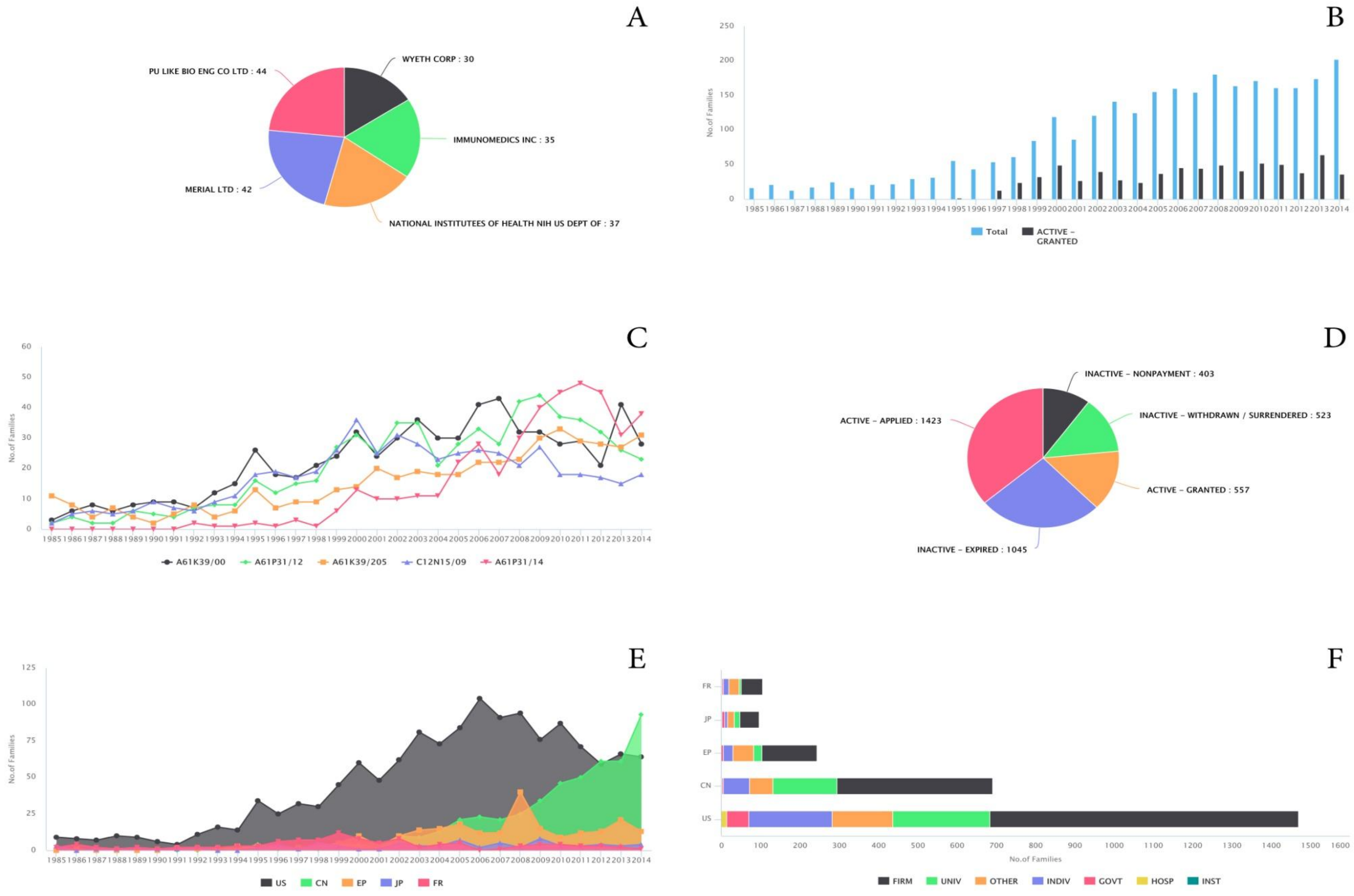
A
WYETH CORP : 30
PU LIKE BIO ENG CO LTD : 44
IMMUNOMEDICS INC : 35
MERIAL LTD : 42
NATIONAL INSTITUTEES OF HEALTH NIH US DEPT OF : 37
B
No.of Families
250
200
150
100
50
0
1985 1986 1987 1988 1989 1990 1991 1992 1993 1994 1995 1996 1997 1998 1999 2000 2001 2002 2003 2004 2005 2006 2007 2008 2009 2010 2011 2012 2013 2014
Total
ACTIVE - GRANTED
C
No.of Families
60
50
40
30
20
10
0
1985 1986 1987 1988 1989 1990 1991 1992 1993 1994 1995 1996 1997 1998 1999 2000 2001 2002 2003 2004 2005 2006 2007 2008 2009 2010 2011 2012 2013 2014
A61K39/00
A61P31/12
A61K39/205
C12N15/09
A61P31/14
D
INACTIVE - NONPAYMENT : 403
ACTIVE - APPLIED : 1423
INACTIVE - WITHDRAWN / SURRENDERED : 523
ACTIVE - GRANTED : 557
INACTIVE - EXPIRED : 1045
E
No.of Families
125
100
75
50
25
0
1985 1986 1987 1988 1989 1990 1991 1992 1993 1994 1995 1996 1997 1998 1999 2000 2001 2002 2003 2004 2005 2006 2007 2008 2009 2010 2011 2012 2013 2014
US
CN
EP
JP
FR
F
FR
JP
EP
CN
US
0 100 200 300 400 500 600 700 800 900 1000 1100 1200 1300 1400 1500 1600
No.of Families
FIRM
UNIV
OTHER
INDIV
GOVT
HOSP
INST

Schistosomiasis

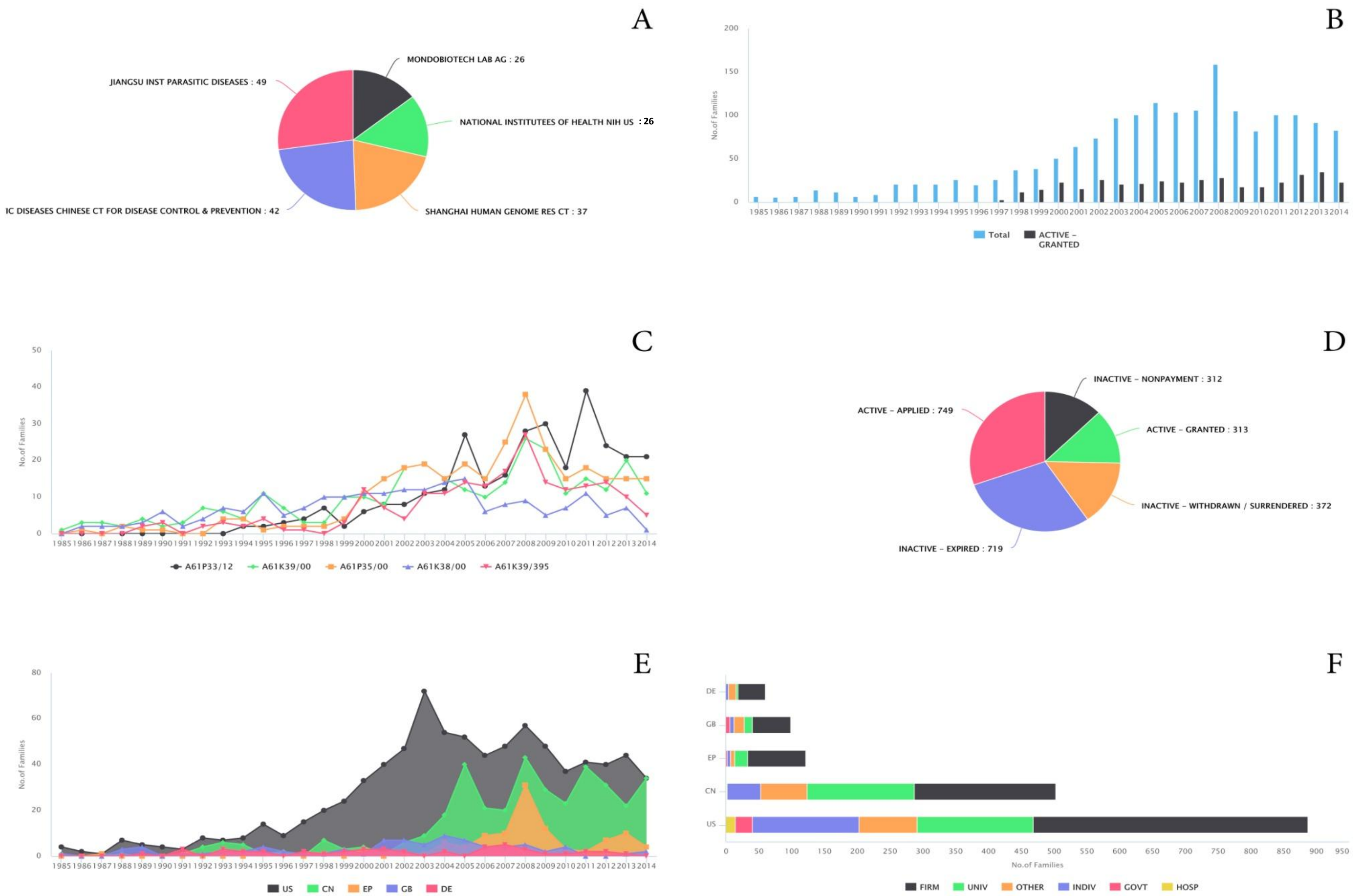

Soil-transmitted helminth infections

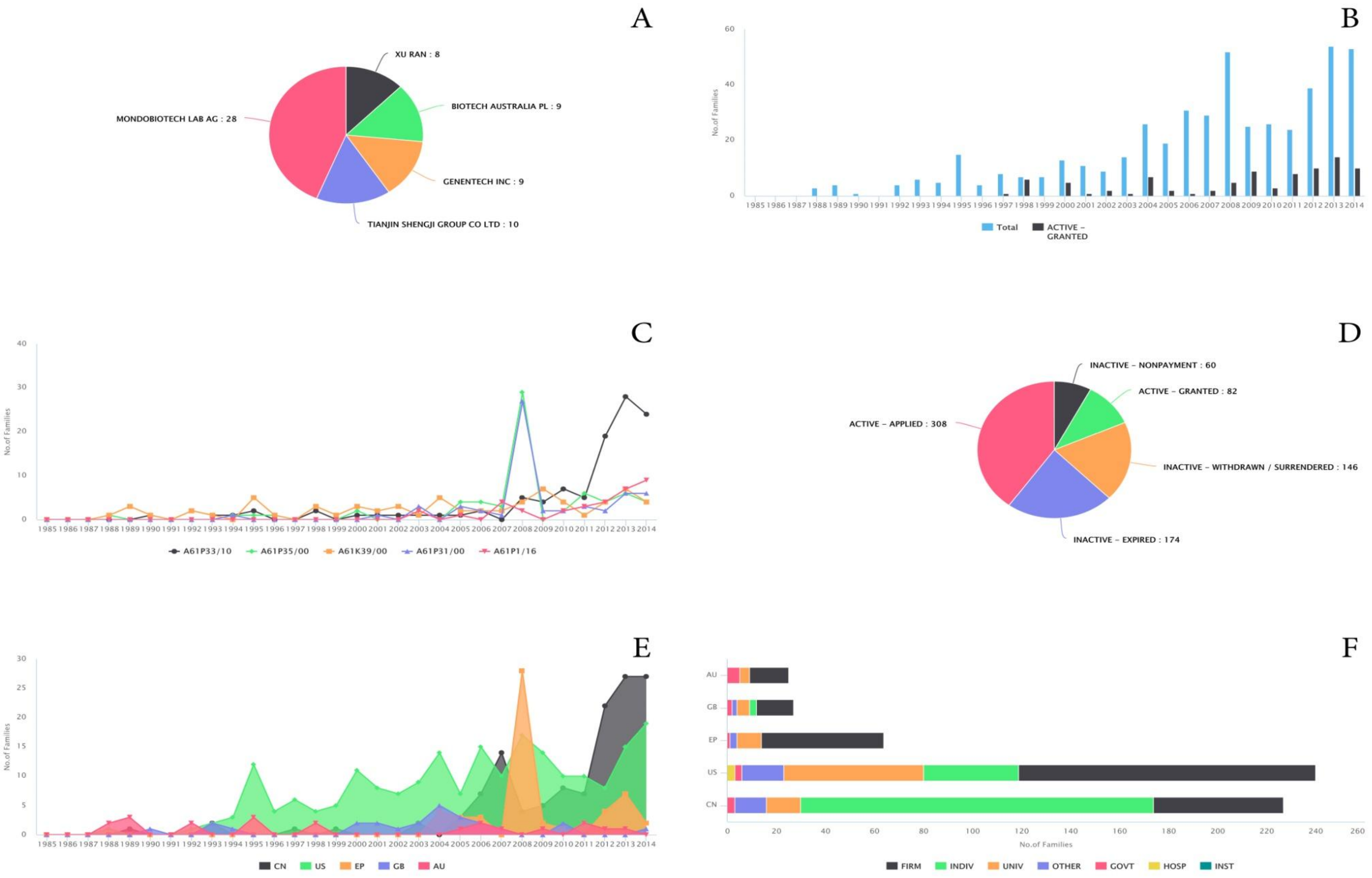

Taeniasis

A

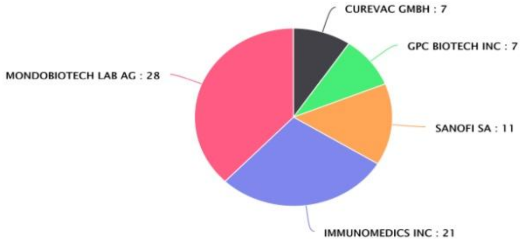

B

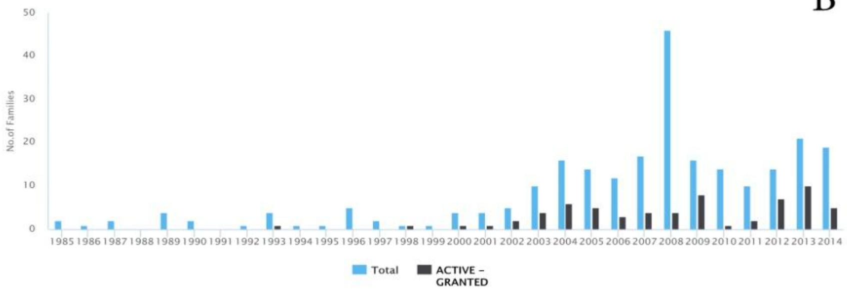

C

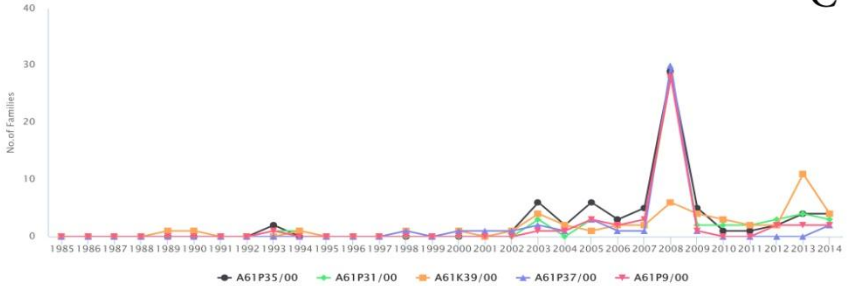

D

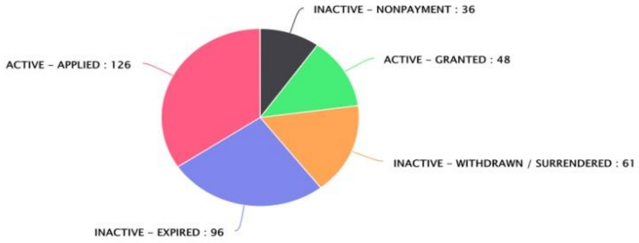

E

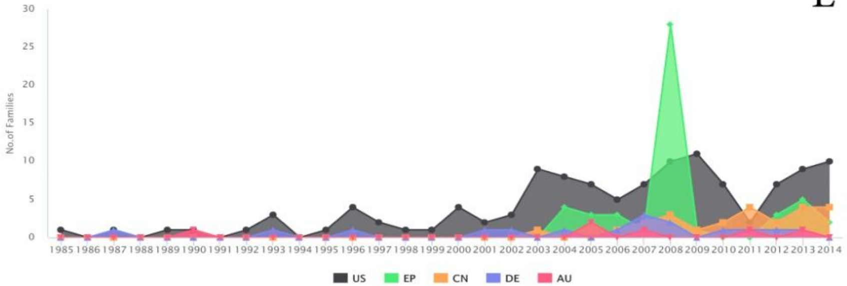

F

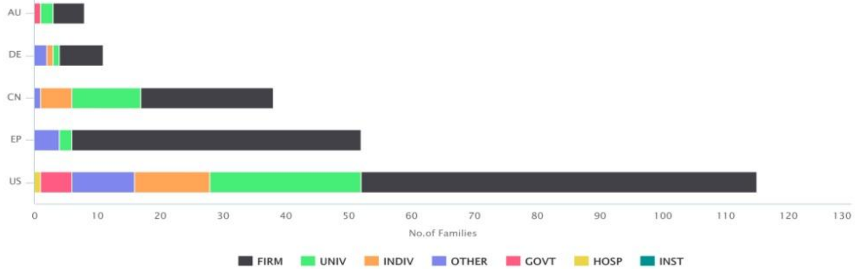

Trachoma

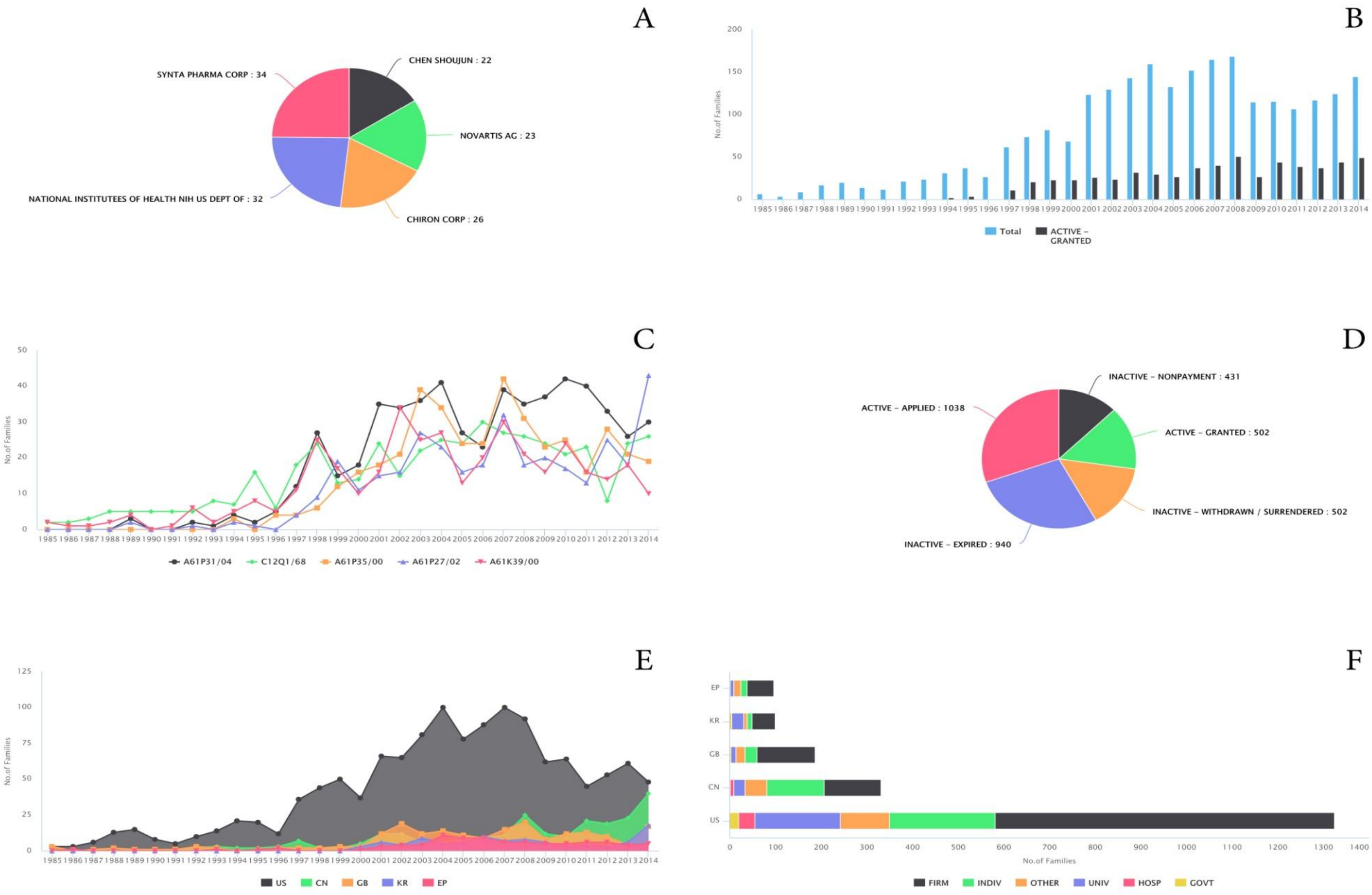

A
CHEN SHOUJUN : 22
SYNTA PHARMA CORP : 34
NOVARTIS AG : 23
NATIONAL INSTITUTEES OF HEALTH NIH US DEPT OF : 32
CHIRON CORP : 26
B
No.of Families
Total
ACTIVE – GRANTED
C
No.of Families
A61P31/04
C12Q1/68
A61P35/00
A61P27/02
A61K39/00
D
INACTIVE – NONPAYMENT : 431
ACTIVE – APPLIED : 1038
ACTIVE – GRANTED : 502
INACTIVE – WITHDRAWN / SURRENDERED : 502
INACTIVE – EXPIRED : 940
E
No.of Families
US
CN
GB
KR
EP
F
EP
KR
GB
CN
US
No.of Families
FIRM
INDIV
OTHER
UNIV
HOSP
GOVT

Yaws

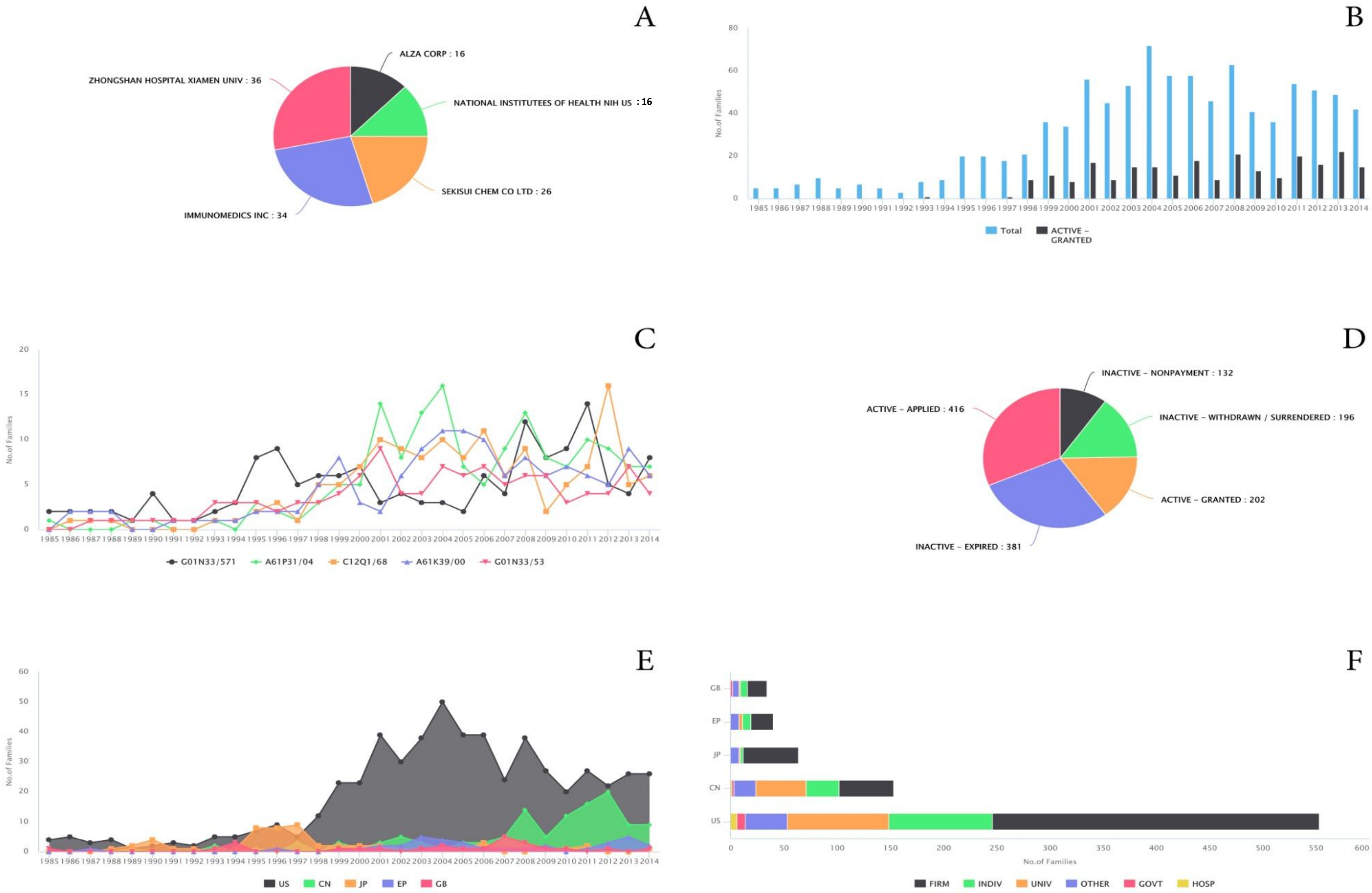
A
ALZA CORP : 16
NATIONAL INSTITUTEES OF HEALTH NIH US : 16
SEKISUI CHEM CO LTD : 26
IMMUNOMEDICS INC : 34
ZHONGSHAN HOSPITAL XIAMEN UNIV : 36
B
No.of Families
Total
ACTIVE – GRANTED
C
No.of Families
G01N33/571
A61P31/04
C12Q1/68
A61K39/00
G01N33/53
D
INACTIVE – NONPAYMENT : 132
INACTIVE – WITHDRAWN / SURRENDERED : 196
ACTIVE – GRANTED : 202
INACTIVE – EXPIRED : 381
ACTIVE – APPLIED : 416
E
No.of Families
US
CN
JP
EP
GB
F
GB
EP
JP
CN
US
No.of Families
FIRM
INDIV
UNIV
OTHER
GOVT
HOSP

Innovative and Intensified Disease Management vs. Preventive Chemotherapy and Transmission Control

G

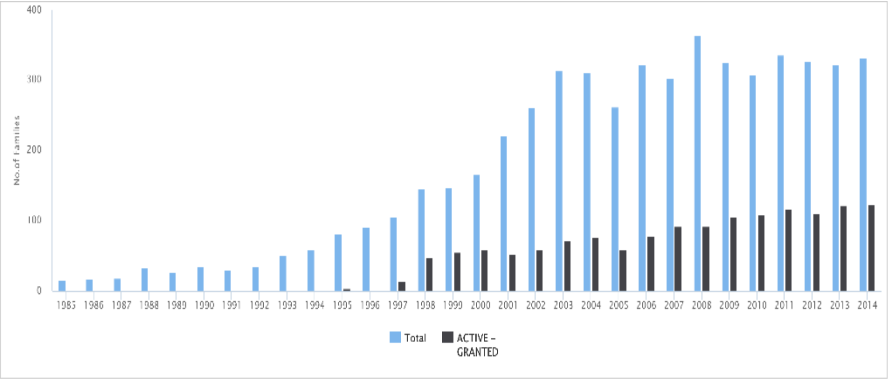

H

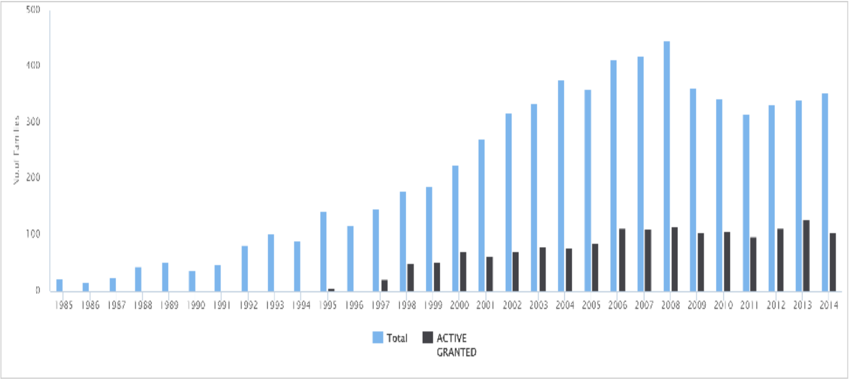

I

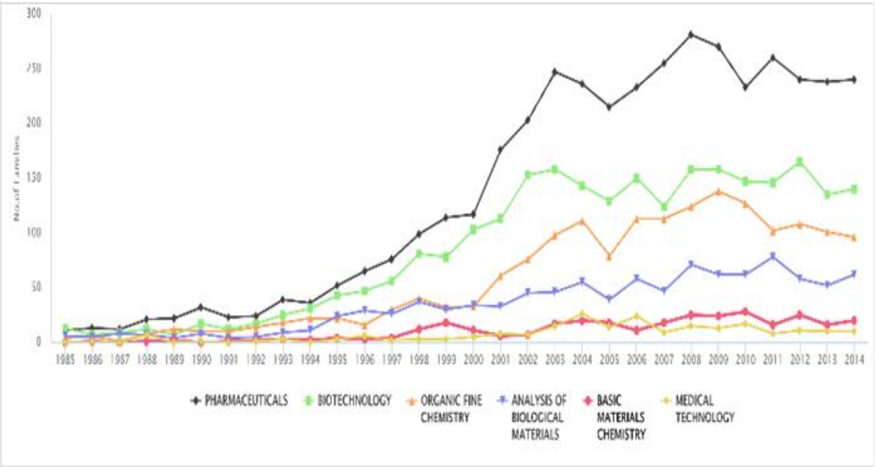

J

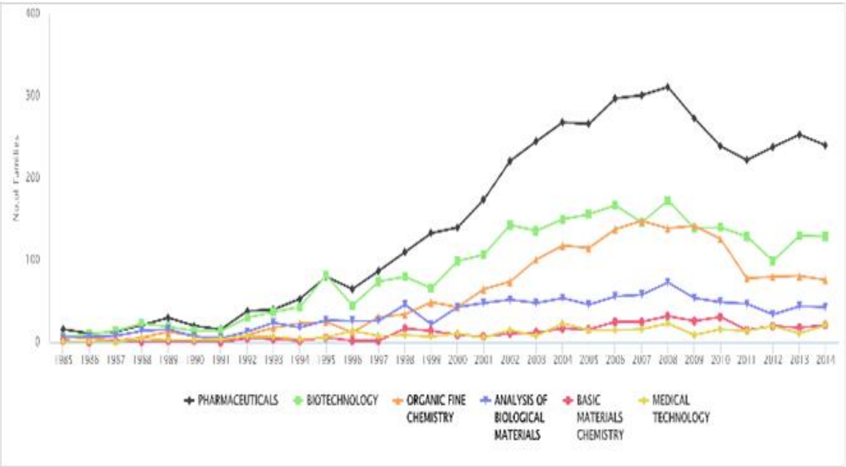

Additional file 2

A. Number of patent families for the top five assignees.

B. Trend of total number of patent families and granted patent families by year.

C. Number of patent families by top five International Patent Classification subgroups, by years.

D. Current legal status of patents. Record numbers referring to the number of patent families.

E. Number of patent families for the top five priority countries, by years.

F. Number of patent families for the top five priority countries by types of assignee (unassigned patent families were not presented).

G. Trend of total number of patent families and granted patent families by year for diseases of Innovative and Intensified Disease Management (Buruli ulcer, Chagas disease, Human African trypanosomiasis, Leishmaniasis, Yaws)

H. Trend of total number of patent families and granted patent families by year for diseases of Preventive chemotherapy and transmission control (Dracunculiasis, Leprosy, Lymphatic filariasis, Onchocerciasis, Schistosomiasis, Soil-transmitted helminths, Trachoma)

I. Technological fields of patent families over time for diseases of Innovative and Intensified Disease Management (Buruli ulcer, Chagas disease, Human African trypanosomiasis, Leishmaniasis, Yaws)

J. Technological fields of patent families over time for diseases of Preventive chemotherapy and transmission control (Dracunculiasis, Leprosy, Lymphatic filariasis, Onchocerciasis, Schistosomiasis, Soil-transmitted helminths, Trachoma)

Additional file 2

ABBREVIATIONS:

firm (firms), indiv (individuals), univ (universities), inst (non-profit institutions), govt (governments) and hosp (hospitals) are assignee types for patents and patent applications.

AU (Australia), BR (Brazil), CN (China), DE (Denmark), EP (European Union-European Patent Office), FR (France), GB (Great Britain), IN (India), JP (Japan), KR (Korean), RU (Russia), SU (Soviet Union (USSR)), US (United States).
