# Supplementary material for: Patent landscape of neglected tropical diseases: an analysis of worldwide patent families
Source: Global Health. 2017 Nov 14;13:82. doi: 10.1186/s12992-017-0306-9 (PMC5686799; doi:10.1186/s12992-017-0306-9)
Supplement: Supplementary file 3 — An inter-disease comparison of patenting activity. (PDF 20 kb) [file 12992_2017_306_MOESM3_ESM.pdf]

| Disease | DALY 2015 | Patent families | Filed /DALY (%) | DALY/Filed |
|---|---|---|---|---|
| Buruli ulcer | **0** | **288** | 0.000% | 0.000 |
| Chagas disease | **236100** | **1453** | 0.615% | 162.491 |
| Dengue | **1892200** | **2262** | 0.120% | 836.516 |
| Dracunculiasis | **0** | **38** | 0.000% | 0.000 |
| Echinococcosis | **600000** | **425** | 0.071% | 1411.765 |
| Food-borne trematodiases | **168500** | **255** | 0.151% | 660.784 |
| Human African trypanosomiasis | **202400** | **161** | 0.080% | 1257.143 |
| Leishmaniasis | **1418900** | **2294** | 0.162% | 618.527 |
| Leprosy | **31000** | **2003** | 6.461% | 15.477 |
| Lymphatic filariasis | **2075000** | **215** | 0.010% | 9651.163 |
| Onchocerciasis | **1135700** | **247** | 0.022% | 4597.976 |
| Rabies | **931600** | **2157** | 0.232% | 431.896 |
| Schistosomiasis | **2613300** | **1486** | 0.057% | 1758.614 |
| Soil transmitted helminthes | **3378300** | **478** | 0.014% | 7067.573 |
| Taeniasis | **503000** | **157** | 0.031% | 3203.822 |
| Trachoma | **279200** | **1874** | 0.671% | 148.986 |
| Yaws | **0** | **853** | 0.000% | 0.000 |
| Cancer | **209359200** | **215149** | 0.103% | 973.089 |
| Lung cancer | **36419500** | **39007** | 0.107% | 933.666 |
| Cardiovascular diseases | **347528900** | **158915** | 0.046% | 2186.885 |
| Malaria | **55769600** | **6067** | 0.011% | 9192.286 |
| HIV/AIDS | **66689500** | **387811** | 0.582% | 171.964 |
| all neglected | **15465200** | **12694** | 0.082% | 1218.308 |

| | |
|---|---|
| MIN | **0** |
| MAX | **347528900** |

DALYs=disability-adjusted life-years.
daly source: https://www.ncbi.nlm.nih.gov/pmc/articles/PMC5388857/table/tbl1/
daly source https://www.ncbi.nlm.nih.gov/pmc/articles/PMC4126350/table/T1/

| ***Norm DALY*** | **All filed*Norm DALY** |
|---|---|
| *0.00000* | 0.000 |
| *0.00068* | 0.987 |
| *0.00544* | 12.316 |
| *0.00000* | 0.000 |
| *0.00173* | 0.734 |
| *0.00048* | 0.124 |
| *0.00058* | 0.094 |
| *0.00408* | 9.366 |
| *0.00009* | 0.179 |
| *0.00597* | 1.284 |
| *0.00327* | 0.807 |
| *0.00268* | 5.782 |
| *0.00752* | 11.174 |
| *0.00972* | 4.647 |
| *0.00145* | 0.227 |
| *0.00080* | 1.506 |
| *0.00000* | 0.000 |
| *0.60242* | 129610.581 |
| *0.10480* | 4087.762 |
| *1.00000* | 158915.000 |
| *0.16047* | 973.600 |
| *0.19190* | 74419.485 |
| *0.04450* | 564.889 |
